# Revision of the Middle Triassic coelacanth *Ticinepomis* Rieppel 1980 (Actinistia, Latimeriidae) with paleobiological and paleoecological considerations

**DOI:** 10.1186/s13358-023-00276-4

**Published:** 2023-09-11

**Authors:** Christophe Ferrante, Heinz Furrer, Rossana Martini, Lionel Cavin

**Affiliations:** 1 Department of Geology and Palaeontology, Natural History Museum of Geneva, CP 6434, 1211 Geneva 6, Switzerland; 2 Department of Earth Sciences, University of Geneva, Rue des Maraîchers 13, 1205 Geneva, Switzerland; 3 Paläontologisches Institut und Museum der Universität Zürich, Karl Schmid-Strasse 4, 8006 Zurich, Switzerland

**Keywords:** Actinistia, Coelacanth, *Ticinepomis*, New species, Monte San Giorgio, Besano Formation, Prosanto Formation, Middle Triassic

## Abstract

Coelacanths form today an impoverished clade of sarcopterygian fishes, which were somewhat more diverse during their evolutionary history, especially in the Triassic. Since the first description of the coelacanth *Ticinepomis peyeri* from the Besano Formation of the UNESCO World Heritage Site of Monte San Giorgio (Canton Ticino, Switzerland), the diversity of coelacanths in the Middle Triassic of this area of the western Paleo-Tethys has been enriched with discoveries of other fossil materials. At Monte San Giorgio, two specimens of *Heptanema paradoxum* and several specimens of the unusual coelacanth *Rieppelia* *heinzfurreri*, have been reported from the Meride Limestone and the Besano Formation, respectively. Another unusual coelacanth, *Foreyia maxkuhni*, and two specimens referred to *Ticinepomis* cf. *T. peyeri* have been described from the isochronous and paleogeographical close Prosanto Formation at the Ducanfurgga and Strel sites (near Davos, Canton Graubünden). In the framework of the revision of the coelacanth material from the Besano Formation kept in the collection of the Paläontologisches Institut und Museum der Universität Zürich (Switzerland), we reviewed the genus *Ticinepomis* on the basis of the holotype and four new referred specimens. Several morphological traits that were little and/or not understood in *T. peyeri* are here clarified. We re-evaluate the taxonomic attribution of the material of *Ticinepomis* cf. *T. peyeri* from the Prosanto Formation. Morphological characters are different enough from the type species, *T. peyeri*, to erect a new species, *Ticinepomis ducanensis* sp. nov., which is shown to be also present in the Besano Formation of Monte San Giorgio, where it is represented by fragmentary bone elements. The recognition of a new coelacanth species indicates that the diversity of this slow-evolving lineage was particularly high in this part of the Western Tethys during the Middle Triassic, especially between 242 and 240 million years ago.

## Introduction

**Fig. 1:**
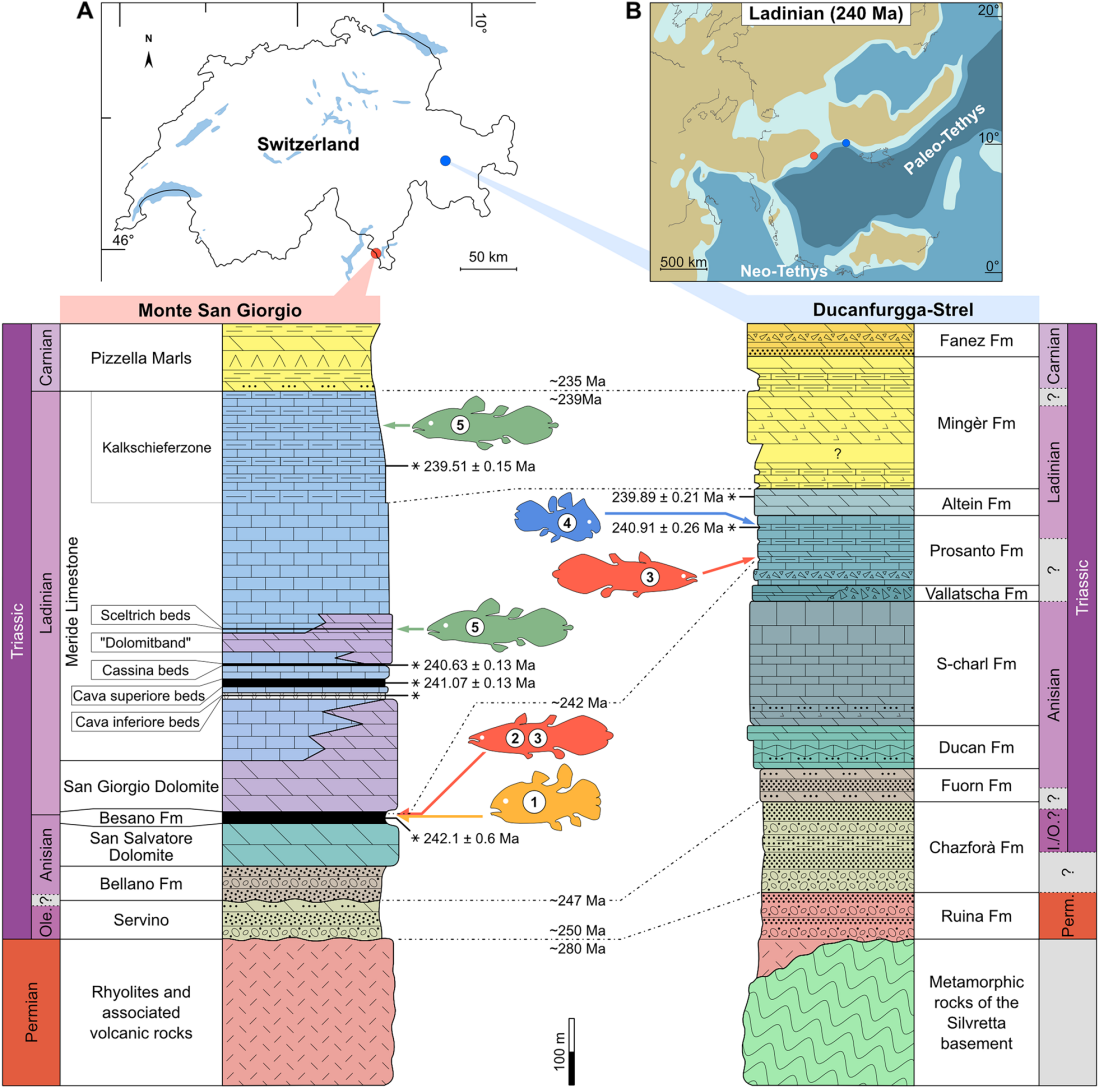
Geographical locations of Middle Triassic coelacanths in Switzerland at Monte San Giorgio and the Ducanfurgga-Strel, and correlations between both stratigraphic sections. **A** Map of Switzerland showing location of sites (top), with (1) *Rieppelia heinzfurreri*, (2) *Ticinepomis peyeri*, (3) *Ticinepomis ducanensis* sp. nov., (4) *Foreyia maxkuhni*, and (5) *Heptanema paradoxum* placed in correlated stratigraphic sections (bottom). **B** Paleogeographic map (modified from Vérard, 2019) showing the location of the two sections in the Ladinian

Middle Triassic coelacanths in Switzerland are known by *Ticinepomis peyeri* (Rieppel, 1980), *Heptanema paradoxum* (Renesto & Stockar, 2018; Renesto et al., 2021), *Foreyia maxkuhni* (Cavin et al., 2017), and *Rieppelia* *heinzfurreri* (Ferrante et al., 2017; Ferrante & Cavin 2023; Rieppel, 1985), recovered from marine deposits of the UNESCO World heritage site of Monte San Giorgio in Canton Ticino and from the localities of Ducanfurgga and Strel in Canton Graubünden (Fig. 1). Apart from the Triassic period, coelacanths are known in Switzerland only in the Lower Jurassic with *Libys callolepis* (Ferrante et al., 2022) recovered from marine deposits near the Teysachaux summit (Canton of Fribourg).

Rieppel (1980) described and named *Ticinepomis peyeri*, a small marine coelacanth species from the upper Besano Formation (former ‘Grenzbitumenzone’, late Anisian/early Ladinian) at Monte San Giorgio (Canton Ticino, southern Switzerland). The description was based on a single specimen (holotype PIMUZ T 3925) represented by a 180 mm-long sub-complete coelacanth preserved as part and counterpart. Five years later, Rieppel (1985) mentioned the presence of another coelacanth taxon from the Besano Formation that he tentatively referred to cf. *Holophagus picenus*. The latter corresponds to a new genus and species of an unusual coelacanth recently named *Rieppelia heinzfurreri* (Ferrante & Cavin, 2023). Beside these specimens, the collection of the Paläontologisches Institut und Museum der Universität Zürich (PIMUZ) contains about 50 undescribed sub-complete and fragmentary specimens of coelacanths from the Besano Formation at Monte San Giorgio, which were found during several excavation campaigns carried out during the twentieth century. More than 30 years after Rieppel’s descriptions, Cavin et al. (2013) referred two coelacanth specimens (PIMUZ A/I 1959 and 2985) from the Middle Triassic Prosanto Formation of the Ducan–Landwasser region near Davos (Canton Graubünden, eastern Switzerland) to *Ticinepomis* cf. *T. peyeri*. In this study, the authors reviewed the holotype of *T. peyeri* (PMIUZ T 3925) and proposed an emended diagnosis of the genus. A few years later, Cavin et al. (2017) described the morphologically unusual coelacanth *Foreyia maxkuhni* from the upper Prosanto Formation. Renesto & Stockar (2018) and Renesto et al. (2021) recorded the occurrence of another species of coelacanth at Monte San Giorgio, *Heptanema paradoxum*, from the Sceltrich beds and the Kalkschieferzone of the Meride Limestone (early/late Ladinian).

This brief review indicates the relatively high diversity of coelacanths that inhabited two restricted basins (Monte San Giorgio and Prosanto), located in close proximity in northwestern Tethys for a short period of about 2 million years spanning the Anisian/Ladinan boundary.

In a new project dealing with revision of the coelacanth material from the Besano Formation housed in the collection of the PIMUZ, four specimens were assigned to *Ticinepomis peyeri* (PIMUZ T 978, 1513, 2651, 2653). In addition, three other specimens (PIMUZ T 585, 2770, 340) were referred to *T.* cf. *peyeri.* Thanks to these new *Ticinepomis* specimens, we clarify several morphological traits that were little and/or not understood in the holotype of *T. peyeri* (PMIUZ T 3925).

We re-evaluate the generic and specific attribution of the two specimens described by Cavin et al. (2013) from the Prosanto Formation (Graubünden, Switzerland) and we found that one specimen (PIMUZ A/I 1959) belongs to the genus *Foreyia* (Cavin et al., 2017). The other specimen (PIMUZ A/I 2985) indeed belongs to the genus *Ticinepomis,* but to a new species, *Ticinepomis ducanensis* sp. nov. This new species is also present at Monte San Giorgio as indicated by a specimen (PIMUZ T 435) described here, which presents the same morphological traits than the specimen from the Prosanto Formation. Here, we point out the specific characters of the new species by describing the two aforementioned specimens.

## Materials and methods

The studied specimens from the Besano Formation at Monte San Giorgio were collected during industrial mining for bituminous shales and by systematic excavations under direction of Bernhard Peyer and Emil Kuhn-Schnyder of the University of Zurich in the twentieth century (Kuhn-Schnyder, 1974; Peyer, 1944). The fossils were found on both the Swiss and Italian sides of Monte San Giorgio, a transnational UNESCO heritage since 2003/2010. Most of the specimens were collected at the Swiss localities Cava Tre Fontane, Valle Stelle, and Miniera Val Porina (Meride/Mendrisio, Canton Ticino, Switzerland) and few at the Italian locality of Cave di Besano (Besano/Porto Ceresio, Province of Varese, Italy). The specimens from the Prosanto Formation were found during systematic excavations under direction of one of the authors (HF) between 1992 and 2020 at the localities Strel and Ducanfurgga (near Davos, Canton Graubünden, Switzerland).

The specimens were mechanically prepared under a binocular, using air tools, thin steel needles, and sand blaster with sodium bicarbonate as abrasive, depending on their fragility. All fossils are hosted in the Paleontological Institute and Museum of the University of Zurich (PIMUZ).

## Geology, stratigraphy, and palaeoenvironment

### Besano Formation

The Besano Formation, first introduced as the ‘Grenzbitumenzone’ by Frauenfelder (1916) and also known as ‘Scisti bituminosi’ or ‘Serpiano Shales’ (Andersson, 1916; De Alessandri, 1910), is the most fossiliferous part of the Monte San Giorgio area in the western Southern Alps in southern Switzerland and northern Italy (Furrer & Vandelli, 2014; Renesto & Stockar, 2018). The 15–16 m-thick sequence consists of 10–30 cm-thick light grey laminated dolomites, finely laminated organic-rich dark dolomites, alternating with thinner black bituminous shales, and some rare and thin layers of volcanic tuffs (e.g., Furrer, 2003; Röhl et al., 2001). The sediments deposited during the latest Anisian-to-earliest Ladinian under mainly anoxic conditions. The deposition interval is estimated at 2 million years, based on Mundil et al. (2010) and Stockar et al. (2012).

The Besano Formation is divided into three informal sub-units (lower, middle, and upper Besano Formation), with individual beds numbered from 3 to 186 during the most important systematic excavation at the site Point 902/Mirigioli under direction of E. Kuhn-Schnyder from 1950 to 1968 (Rieber, 1973; Röhl et al., 2001). However, interesting fossils were also collected in galleries of the former mines in the black shales of the middle Besano Formation (‘Cava Tre Fontane’, ‘Valle Stelle’, and ‘Miniera Val Porina’ on the Swiss side, and ‘Cave di Besano’ on the Italian side (Stockar et al., 2013, fig. 1A). The individual beds with old names by the miners can be correlated with the standard profile at Point 902/Mirigioli, where the lowest well-laminated dolomite layer marks the base of the Besano Formation. Unfortunately, the outcrop of this standard profile is now covered by debris and vegetation.

The lower Besano Formation (beds 3–53) consists of well-laminated dolomitic mudstone and stromatolites with a few mainly disarticulated vertebrate fossils that were deposited in the sub- to intertidal environment of a restricted lagoon (Röhl et al., 2001). It is dated as *Reitziites reitzi* Ammonoid Zone (late Anisian). The middle Besano Formation (beds 54 to 132) is mainly formed by dolomitic biomicrites (mud- to packstone) and black shales with high Total Organic Carbon content up to 43.7% (Bernasconi, 1994). These beds were deposited in an intraplatform basin with a low-energy depositional environment and are marked by a peak of taxic diversity of bivalves, ammonoids, actinopterygians, actinistians, chondrichthyans, conodonts, and ichthyosaurs, suggesting some connection to the open sea with normal marine surface water conditions (Furrer, 2003). Bed 71 corresponds to a volcanic ash layer, which gave a minimum U–Pb age of 242.1 ± 0.6 Ma (Mundil et al., 2010). The middle Besano Formation (beds 133–186) belongs to the *Nevadites secedensis* Ammonoid Zone (latest Anisian). The upper Besano Formation is composed mainly of irregularly laminated dark dolomitic mudstone, packstone, and wackestone deposited in subtidal environment with low-energy and occasionally high-energy influences (Röhl et al., 2001). Based on increasing radiolarians, Bernasconi (1994) suggested a better connection to the open sea. However, Furrer (1995) and Röhl et al. (2001) proposed that the basin progressively transformed into a lagoonal environment probably separated from the open sea by growing carbonate platforms. The Anisian–Ladinian boundary is located between beds 149 and 150 with onset of the *Eoprotrachyceras curionii* Ammonoid Zone (earliest Ladinian; Brack et al., 2005, fig. 7).

During the Middle Triassic, the area of Monte San Giorgio, as part of the western Southern Alps province, was located at a northern intertropical latitude of about 15–18° in the north western Tethys with shallow carbonate platforms, small restricted, and open marine basins (Fig. 1B; Muttoni et al., 2004). In the late Anisian, the climate of the western area of the Tethys was humid as indicated by palynomorph associations (Preto et al., 2010). The Middle Triassic basin of Monte San Giorgio, connected to the western Paleo-Tethys, was located on a passive continental margin that was gradually submerged by a long-term transgression coming from the east (Stockar et al., 2013). The sedimentary sequence, then located in a marginal zone, started in the Olenekian, Early Triassic (Fig. 1A). The Middle Triassic carbonates (dolomite and limestone) with partly bituminous marl- and claystone and volcanoclastic layers were deposited under temporarily dysoxic-to-anoxic bottom water conditions and had recorded major thanatocoenosis in various depositional environments (e.g., Furrer, 1995; Stockar et al., 2012, 2013).

The basin during deposition of the Besano Formation was estimated to have a diameter of at least 10 km and a depth of 100–150 m (Furrer, 1995). However, Bernasconi, (1994), based on tectonic studies by Bertotti (1991), suggested that the Monte San Giorgio basin was connected to the basin of the Perledo-Varenna Formation, east of Lake Como (Italy), forming together a single large basin of about 20 km in diameter. According to Stockar et al. (2012) and López-Arbarello et al. (2014), the Varenna Limestone possibly corresponds to the uppermost Besano Formation (earliest Ladinian), and the lower part of the Meride Limestone (early Ladinian), while the Perledo Member possibly correlates with the uppermost Meride Limestone (‘Kalkschieferzone’, late Ladinian). The accumulation and extraordinary state of preservation of the fossils in the Besano Formation can be explained by the combination of a low sedimentation rate, 1–5 mm per thousand years, and a high supply of organic matter associated with anoxic bottom water conditions (Furrer, 1995).

### Prosanto Formation

The Prosanto Formation is part of strongly deformed Triassic and Permian sediments of the Austroalpine Silvretta nappe (Bürgin et al., 1991; Furrer et al., 1992). Embedded in Middle Triassic-aged, light grey, shallow water carbonates, the Prosanto Formation comprises a 100 to 200 m-thick sequence of dark limestone, shales, and dolomite. It extends for more than 20 km in East–West and South–North direction as a lenticular intercalation in shallow water dolomites (overlying the Vallatscha and underlying the Altein formations; Fig. 1A). Since 1989, systematic prospecting and excavations by a team of Zurich University staff and volunteers, directed by one of us (H.F.), provided a rich fauna of well-preserved vertebrates, including coelacanths, and invertebrate fossils from this fossil lagerstätte (Cavin et al., 2013, 2017; Furrer, 2019). Cavin et al. (2013) described the first two coelacanths as *Ticinepomis* cf. *T. peyeri*.

Lithostratigraphy and fossils of the Prosanto Formation share many similarities with the classic Middle Triassic fossil site of the Monte San Giorgio area in the southern Alps (Anisian/Ladinian), corroborated by U/Pb zircon ages from volcanic ash layers in the fossiliferous beds of the upper Prosanto Formation (240.91 ± 0.26 Ma) and the overlying Altein Formation (239.89 ± 0.21 Ma; Furrer et al., 2008). It suggests a well-based correlation of the upper Prosanto Formation with the lower Meride Limestone (*“Eotrachyceras” gredleri* Ammonoid Zone, Early Ladinian; Furrer et al., 2008; Mundil et al., 2010; Stockar et al., 2012). The exact bio- and chronostratigraphic position of site DF 10 in the strongly deformed middle part of the Prosanto Formation is not known. A poorly preserved ammonoid, not identifiable to genus or species level, found in 2017, suggests a Late Anisian age (written communication by Hans Rieber, 2019). That allows a possible correlation of the middle part of the Prosanto Formation with the upper and middle Besano Formation (Fig. 1A).

The rich and well-preserved fish and reptile fauna of the Prosanto Formation suggests a deposition in stagnant abiotic, probably anoxic bottom water conditions of a small intraplatform basin. Small plankton feeding fishes such as *Habroichthys* and large predatory fishes such as *Saurichthys* together with the rare sauropterygian reptiles lived in the surface water. Medium-sized fish preying on hard-shelled bivalves and crustaceans, but also calcareous algae must have lived at the border of the basin in a shallow water environment. Echinoderms and cephalopods are very rare, suggesting euryhaline surface water of a restricted basin (Furrer, 2019). The paleogeographic position of the Prosanto basin must have been some hundred kilometres northeast of the Monte San Giorgio basin (Fig. 1B). However, the exact position of the Silvretta nappe as part of the westernmost Austroalpine nappes is not known (Furrer, 2019; Pfiffner, 2014).

## Systematic status of the *Ticinepomis* cf. *T. peyeri* specimens from the Prosanto Formation (Middle Triassic), Ducan and Landwasser regions

In 2013, Cavin et al. (figs 4, 5, 6 and 7) described two specimens of coelacanths from the Middle Triassic Prosanto Formation of the Ducan and Landwasser regions (Canton Graubünden, Switzerland). The material is represented by a fragmentary caudal fin (PIMUZ A/I 1959) from the upper Prosanto Formation at the locality Strel (Landwasser region, southwest of Davos) and a sub-complete specimen (PIMUZ A/I 2985) from the middle Prosanto Formation at the locality Ducanfurgga (Ducan region, south of Davos). By comparison with the holotype of *Ticinepomis peyeri* (PIMUZ T 3925) described by Rieppel (1980) from the Middle Triassic of Monte San Giorgio (Ticino, Switzerland), Cavin et al. (2013) referred with caution both specimens to *Ticinepomis* cf. *T. peyeri*. Considering the present redescription of the holotype of *Ticinepomis peyeri* and following the discovery of *Foreyia maxkuhni* (Cavin et al., 2017), we re-evaluate here the generic and specific attribution of the material described by Cavin et al. (2013).

### Specimen PIMUZ A/I 1959

**Fig. 2:**
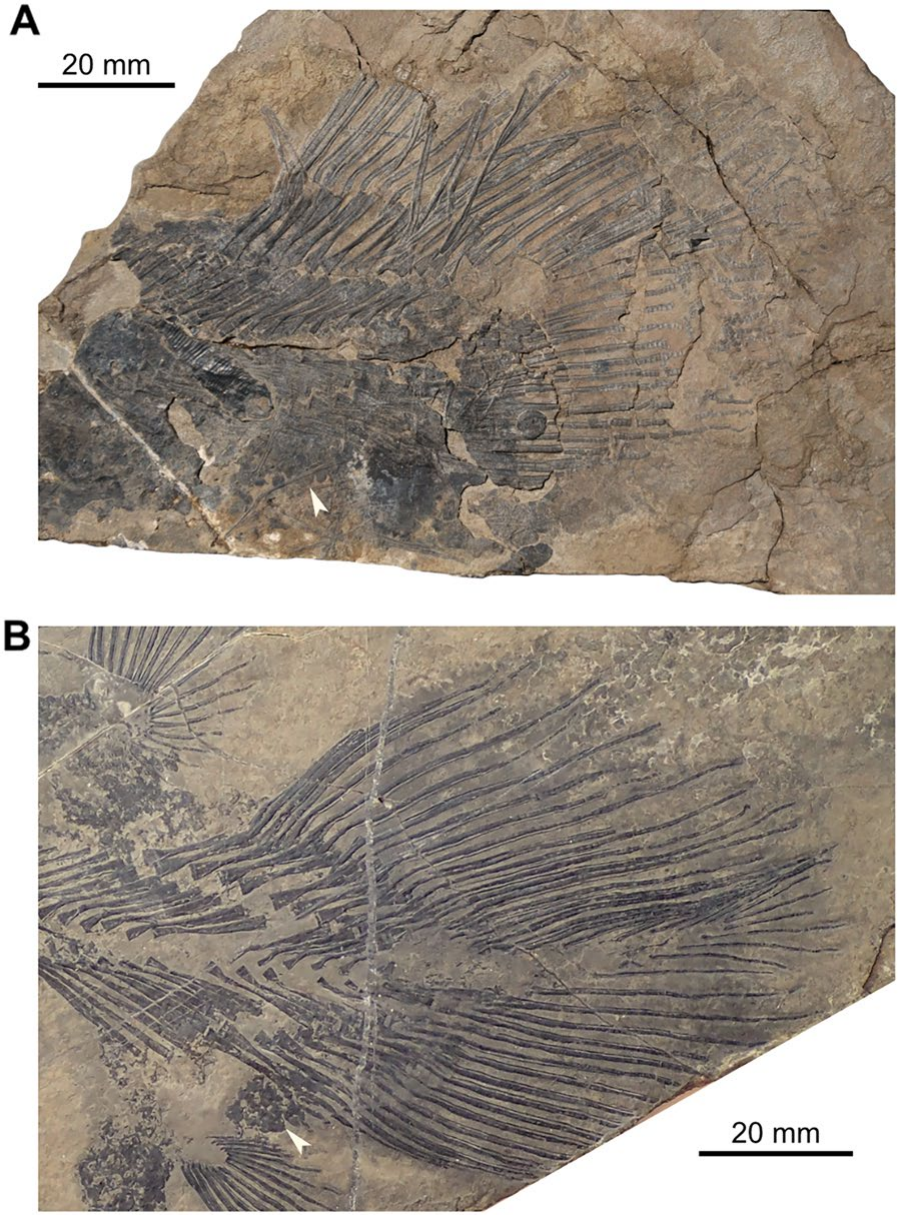
Caudal fins of *Foreyia maxkuhni*. **A** Specimen PIMUZ A/I 1959 and **B** holotype PIMUZ A/I 4620 (both from the upper Prosanto Formation; white arrow-heads indicate round scales)

The caudal fin (PIMUZ A/I 1959) includes about 15 neural and 15 haemal arches (Fig. 2A; Cavin et al., 2013, fig. 7). There are 19 and 14 rays (minimal counting) on the dorsal and ventral lobes, respectively, with a one-to-one relationship with the supporting radials. The supplementary caudal fin lobe is supported by circa 8 small rays. According to Cavin et al. (2013), the structure of the caudal fin (PIMUZ A/I 1959) is consistent with the caudal fin of the holotype of *Ticinepomis peyeri* (PIMUZ T 3925), which has 18 rays (15 segmented plus 3 unsegmented rays) in both lobes according to the description of Rieppel (1980). A new observation of the holotype of *T. peyeri* (PIMUZ T 3925) and the attribution of a new specimen (PIMUZ T 2651) to this species show that there are in fact 15 rays in both lobes of the caudal fin of *T. peyeri*. In addition to this putative meristic resemblance, which is now dismissed, Cavin et al. (2013) noticed two main differences between the caudal fin (PIMUZ A/I 1959) and that of the holotype of *T. peyeri* (PIMUZ T 3925). First, the rays of the caudal fin (PIMUZ A/I 1959) are devoid of denticles, while the anterior most rays of *T. peyeri* (PIMUZ T 2651 and 3925) are ornamented with small and sharp denticles. Second, the scales (Fig. 2A) found on PIMUZ A/I 1959 have a circular pattern and are ornamented with faint ridges and no denticles, unlike in *T. peyeri* that bear oval scales ornamented with a pack of elongated ridges (holotype PIMUZ T 3925) surrounding, in some cases, a median larger ridge (PIMUZ T 2651 and 2653). The caudal fin of *Foreyia maxkuhni* (Fig. 2B; Cavin et al., 2017, figs S2-3), discovered few years later in the same beds of the upper Prosanto Formation at the locality Ducanfurgga, shares characteristics with the caudal fin (PIMUZ A/I 1959). The caudal fin of *Foreyia maxkuhni* is composed of 14 radials (contra 16 in Cavin et al., 2017) and 17 rays, and 13 radials (contra 14 in Cavin et al., 2017) and 16 rays in the dorsal and ventral lobe, respectively, which is consistent with the caudal fin (PIMUZ A/I 1959) that exhibits 14 radials with circa 17 rays, and a minimal count of circa 11 radials and 11 rays, respectively. Similarly to PIMUZ A/I 1959, the posterior tip of the supplementary caudal fin lobe of *Foreyia maxkuhni* is composed of approximately 8 rays (Cavin et al., 2017) including 4 anterior rays on both the dorsal and ventral parts. Additionally, the supplementary lobe of the caudal fin (PIMUZ A/I 1959), which is difficult to observe, does not extend outward from the caudal fin (Fig. 2A) but is included in the fin profile, as in *Foreyia maxkuhni* (Fig. 2B). *F. maxkuhni* has 18 haemal arches, which is close to the number of haemal arches (at least 15) present in PIMUZ A/I 1959. The peculiar scales observed on PIMUZ A/I 1959 (Fig. 2A) are similar to the round scales ornamented with two or four spines present in *F. maxkuhni* (Fig. 2B; Cavin et al., 2017). The absence of denticles on the fin rays of PIMUZ A/I 1959 is, however, in opposition to the condition seen in *F. maxkuhni* that has minute denticles on most of its caudal fin rays (Cavin et al., 2017). Cavin et al. (2013) assumed that the absence of denticles was due to the mode of preservation. It is however unlikely that the denticles were lost during taphonomic processes, because PIMUZ A/I 1959 is preserved in a marlstone and was most probably not transported a long distance before burial. This absence of denticles in PIMUZ A/I 1959 can be caused either by a younger ontogenetic stage, or by sexual dimorphism. Therefore, according to the aforementioned observations, the caudal fin (PIMUZ A/I 1959) referred by Cavin et al. (2013) to *Ticinepomis* cf. *T. peyeri* is reattributed here to *Foreyia maxkuhni*.

### Specimen PIMUZ A/I 2985

The sub-complete specimen (PIMUZ A/I 2985) is well preserved but only visible in ventral view, making it difficult to assign an accurate specific identification as most of the diagnostic characters of coelacanths are observed on the skull roof and on the cheek (Cavin et al., 2013, figs 4, 5). Nevertheless, Cavin et al. (2013) found that this specimen shares many characters with the holotype of *Ticinepomis peyeri* (PIMUZ T 3925), such as: (1) the length of the otico-occipital portion being less than half the length of the ethmosphenoid portion; (2) a premaxilla with 4 teeth; (3) a dentary with a pronounced ventral angle midway along its length; (4) a large splenial with a curved shape; (5) a splenial deeper than the dentary; (6) an ovoid-shaped opercle; (7) a dorsally expanded cleithrum; (8) a horizontal extension of the clavicle; (9) a basal plate of the anterior dorsal fin approximately triangular; (10) expanded distal extremities of the rays of the anterior dorsal fin, and (11) the presence of denticles on the anterior rays of anterior dorsal fin. According to Cavin et al. (2013), PIMUZ A/I 2985 differs only by its larger size, reaching an estimated total length of circa 615 mm, while the holotype of *T. peyeri* (PIMUZ T 3925) is circa 180 mm in length. This difference in size is here regarded as a specific variation and not as an individual variation, because in both PIMUZ T 3925 and PIMUZ A/I 2985, the basal plates are fully ossified, which is a feature observed in adult coelacanths (e.g., Schultze, 1980; Witzmann et al., 2010).

**Fig. 3:**
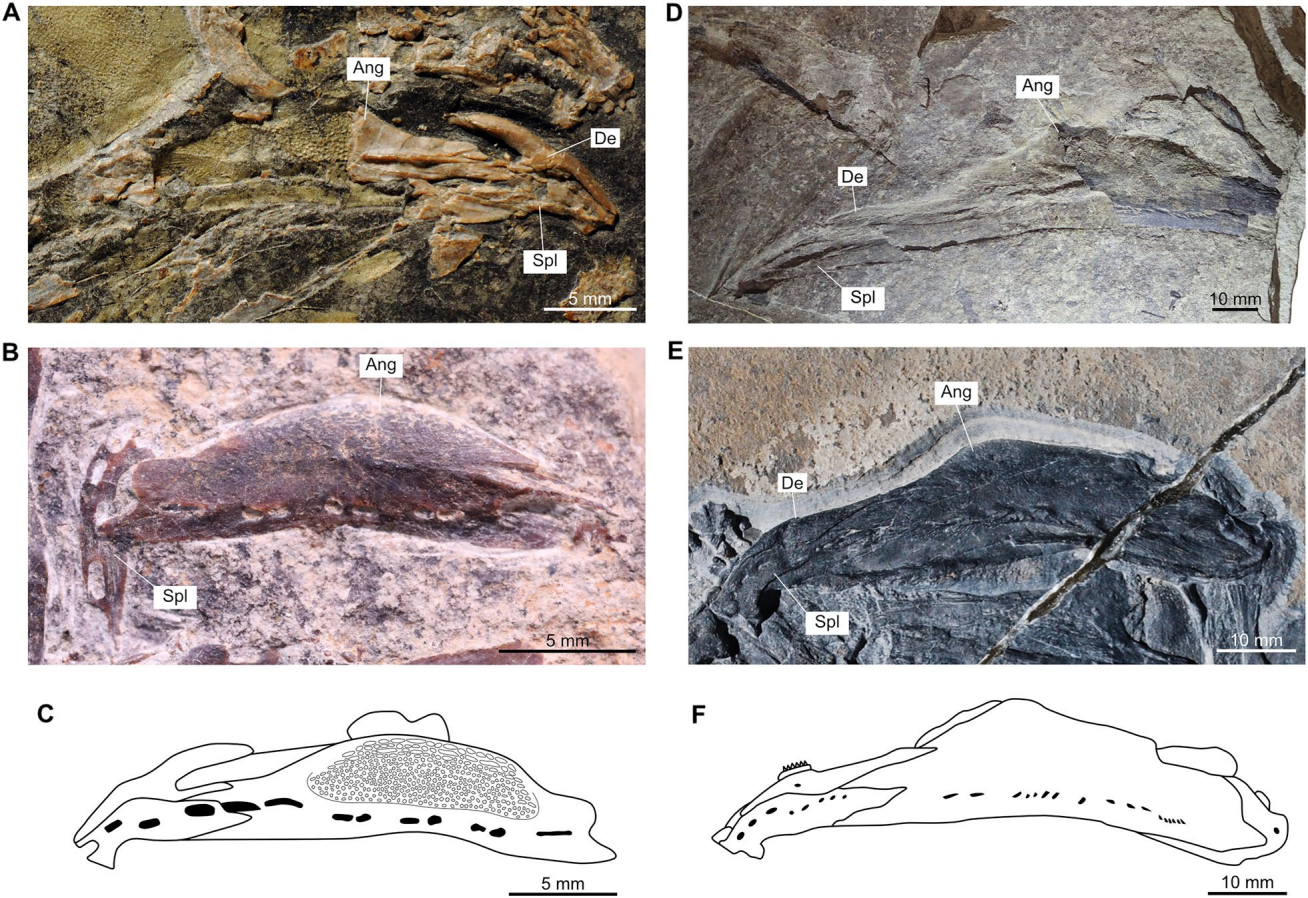
Lower jaws of *Ticinepomis* spp. **A** Holotype (counterpart) PIMUZ T 3925b of *Ticinepomis peyeri*. **B** Specimen PIMUZ T 978 of *T. peyeri*. **C** Reconstruction of the lower jaw of *T. peyeri* (based on the holotype PIMUZ T 3925 and specimen PIMUZ T 978).** D** Specimen PIMUZ T 435 of *T. ducanensis* nov. sp. **E** Holotype PIMUZ A/I 2985 of *T. ducanensis* sp. nov. **F** Reconstruction of the lower jaw of *T. ducanensis* sp. nov. (based on the holotype PIMUZ A/I 2985 and the specimen PIMUZ T 435)

Besides this difference between the sub-complete specimen (PIMUZ A/I 2985) and *Ticinepomis peyeri*, we found other differences and similarities, especially in the lower jaw. In *T. peyeri*, the angular is strongly ornamented with wavy linear elongated tuberculation to smaller roundish irregular-shaped and coarse tubercles (Fig. 3A–C). Conversely, the angular of PIMUZ A/I 2985 is smooth and is only ornamented with faint ridges (Cavin et al., 2013, fig. 5) with no tubercular ornamentation (Fig. 3E, F). According to Cavin et al. (2013), the mandibular sensory canal opens within the angular of PIMUZ A/I 2985 through 6–7 pores, which would be similar to the situation in *T. peyeri*. However, a re-evaluation of PIMUZ A/I 2985 and a comparison with another referred material (PIMUZ T 435) from Monte San Giorgio reveal that there are much more pores, at least 15, which are proportionally smaller and differently disposed compared to *T. peyeri*. Beside this difference, the angular of *T. peyeri* compared to those of PIMUZ A/I 2985 and PIMUZ T 435 are similar in that they are both relatively shallow and parallel-sided, which is peculiar among coelacanths. The splenial is proportionally similar in size and strongly curved anteriorly downwards in specimens of *T. peyeri*, PIMUZ A/I 2985 ands PIMUZ T 435 (Fig. 3). On the anterior border of the splenial, the notch for the symphyseal pore is present in PIMUZ A/I 2985, but is less deep than in *T. peyeri* (Fig. 3C, F). Within the splenial of PIMUZ A/I 2985, the mandibular sensory canal opens anteriorly through three large pores and then posteriorly through five pores (Fig. 3E, F; Cavin et al., 2013), while in *T. peyeri* (PIMUZ T 978), there are only three large pores of similar size that are preceded by a larger one located at the suture between the splenial and the angular (Fig. 3B,C). In PIMUZ A/I 2985, the dentary is developed as an elongated and narrow splint-like bar (Fig. 3E, F), while in *T. peyeri*, it is strongly hook-shaped (Fig. 3A and C).

Although the axial skeleton of PIMUZ A/I 2985 is incomplete and the exact number of neural arches is unknown, there were probably less than 50 neural arches and at least 12 haemal arches. This number of neural arches is close to that of *T. peyeri* which has 47 neural arches and 15–16 haemal arches. The anterior dorsal fin of PIMUZ A/I 2985 is composed of 10 rays including two small rays anteriorly that are followed by 8 very long rays decreasing in length backward (Cavin et al., 2013). In *T. peyeri*, the anterior dorsal fin contains 8 elongated rays with one or two considerably smaller rays anteriorly. Therefore, there is no important variation in the number of rays in the anterior dorsal fin between the holotype of *T. peyeri* (PIMUZ T 3925) and PIMUZ A/I 2985. Finally, in both *T. peyeri* and PIMUZ A/I 2985, the rays of all fins, except the anterior first dorsal, are all not expanded to some degree but are slender as usually in coelacanths. It is worth noting that Cavin et al. (2013) followed Forey (1998), who wrote in his emended diagnosis of *Ticinepomis* that “all of the fin rays of the median fins are expanded to some degree”, a condition which is here rejected for this taxon. Therefore, in agreement with Cavin et al. (2013), the sub-complete specimen (PIMUZ A/I 2985) shares sufficiently features allowing to attribute it to the genus *Ticinepomis*. Nevertheless, some particular morphological traits of PIMUZ A/I 2985, namely in the lower jaw and its larger size, indicate that it belongs to a distinct species than the type species.

According to previous comments, PIMUZ A/I 2985 shares many characters with the genus *Ticinepomis*, namely: (1) the length of the otico-occipital portion less than half the length of the ethmosphenoid portion; (2) a premaxilla with 4 teeth; (3) a shallow and parallel-sided lower jaw; (4) a large splenial with an anterior portion curved downward; (5) presence of a symphyseal pore on the splenial; (6) a dentary with a pronounced ventral angle midway along its length; (7) a basal plate of the anterior dorsal fin approximately triangular; (8) an anterior dorsal fin composed of 8 long rays plus two smaller anterior rays; (9) the presence of denticles on the anterior rays of the anterior dorsal fin; (10) a robust dorsally expanded cleithrum; (11) a horizontal extension of the clavicle; (12) an ovoid-shaped opercle; (13) a prootic extending backward forming a posterior wing; (14) a low number (less than 50) of neural arches.

Nevertheless, PIMUZ A/I 2985 differs from the type species *T. peyeri* by some specific morphological characters, namely: (1) a small premaxilla; (2) an angular with many (at least 15) small and irregularly sized pores; (3) no tubercular ornament on the angular that is smooth with some faint ridges; (4) a splenial with 8 pores, including three anterior large pores plus five posterior small pores; (5) a smaller symphyseal pore on the splenial (6) a dentary developing as an elongated and narrow splint-like bar; (7) a basal plate of the anterior dorsal fin with a straight ventral margin, an anterodorsal margin dug by a notch and no posteroventral spine; (8) the two anterior processes of the basal plate of the posterior dorsal fin form a larger angle (50°); (9) more robust pelvic bones; (10) its larger body size reaching an estimated length of 615 mm.

Therefore, PIMUZ A/I 2985 described and referred by Cavin et al. (2013) to *Ticinepomis* cf. *T. peyeri* belongs to the genus *Ticinepomis* Rieppel, 1980 but not to the species *T. peyeri*, and it should be included as a new species of *Ticinepomis ducanensis* sp. nov.

## Systematic paleontology

**Sarcopterygii** Romer 1955

**Actinistia** Cope 1871

**Latimerioidei** sensu Toriño et al., 2021

**Latimeriidae** sensu Toriño et al., 2021

**Ticinepomiinae** Ferrante & Cavin, 2023

***Ticinepomis*** Rieppel, 1980

**Diagnosis** (emended)

Latimeriidae coelacanths characterised by the following unique combination of characters: anterior and posterior parietals of similar length; supraorbitals as wide as parietals; posterior margin of the skull roof straight; preorbital present; postorbital reduced to a narrow tube surrounding the sensory canal only; lachrymojugal with a posterior triangular shape; splenial with an anterior portion curved downward; splenial forming a symphyseal pore; short body with less than 50 neural arches; ossified lung absent; lobe of the pectoral fin poorly developed; first rays of the anterior dorsal smaller than the posterior rays; denticles on the fin rays of the anterior dorsal fin and the caudal fin.

***T. peyeri*** Rieppel, 1980

**Diagnosis** (emended)

*Ticinepomis* species of small size characterised by the following unique combination of characters: premaxilla large; angular with eight round to oval pores plus one pore located between the angular and the splenial; splenial with three large pores for the mandibular sensory canal; splenial with a small symphyseal pore; dentary hook-shaped with the dorsal process more developed than the ventral process; dermal bones ornamented with wavy linear elongated tuberculation to smaller roundish irregular-shaped and coarse tubercles; basal plate of the anterior dorsal fin with a concave ventral margin and a posteroventral spine; basal plate of the posterior dorsal fin with two anterior processes forming an angle of 40°; pelvic bones with narrow processes.

### Measurements and meristic

Total body length: 180 mm; d1.f*= 9–10; d2.f = 22–23; pect.f = 17; pelv.f = 13; ana.f = 22; cau.f = 15/14–15; n.a = 47; h.a = 19–21*

### Holotype

PIMUZ T 3925, a sub-complete specimen of 180 mm long preserved as part and counterpart; Point 902/Mirigioli, Meride (Canton Ticino, Switzerland); bed unknown, upper Besano Formation, *E. curionii* Ammonoid Zone, earliest Ladinian (Middle Triassic).

### Referred material

PIMUZ T 978, disarticulated and partial specimen showing some bones of the skull including angulars, prearticular, splenial, some bones of the cheek, a lachrymojugal, opercles, supraorbitals, cleithra, bones of the branchial apparatus including a ceratohyal, many small tooth plates, neural arches, and two partial pterygoids; Point 902/Mirigioli, Meride (Canton Ticino, Switzerland); bed unknown, middle/upper Besano Formation, *N. secedensis/E. curionii* Ammonoid Zone, latest Anisian/earliest Ladinian (Middle Triassic).

PIMUZ T 1513, specimen showing a complete neurocranium including the parasphenoid, basisphenoid, otic shelf with prootic and basioccipital, some poorly preserved bones of the skull including the parietals, a fragmented opercle, and some scattered bones of the axial skeleton plus one scale of the occipital region; Point 902/Mirigioli, Meride (Canton Ticino, Switzerland); bed 91, middle Besano Formation, *N. secedensis* Ammonoid Zone, latest Anisian (Middle Triassic).

PIMUZ T 2651, partial specimen of 160 mm length (estimation) preserved on part and counterpart showing the axial skeleton (about 120 mm long) and a partial skull including the postparietal shield (12 mm long) and some other bones; Cave di Besano, Porto Ceresio (Province of Varese, Italy); bed unknown, middle Besano Formation, *N. secedensis* Ammonoid Zone, latest Anisian (Middle Triassic).

PIMUZ T 2653, disarticulated and partial specimen preserved on part and counterpart; Miniera Val Porina, Meride (Canton Ticino, Switzerland); bed 113, middle Besano Formation, *N. secedensis* Ammonoid Zone, latest Anisian (Middle Triassic).

### Locality and horizon

Point 902/Mirigioli, Meride (Canton Ticino, Switzerland); middle and upper Besano Formation, *Nevadites secedensis* and *Eoprotrachyceras curionii* Ammonoid Zones, latest Anisian-to-earliest Ladinian (Middle Triassic).

## Description of *Ticinepomis peyeri*

### Generalities

**Fig. 4:**
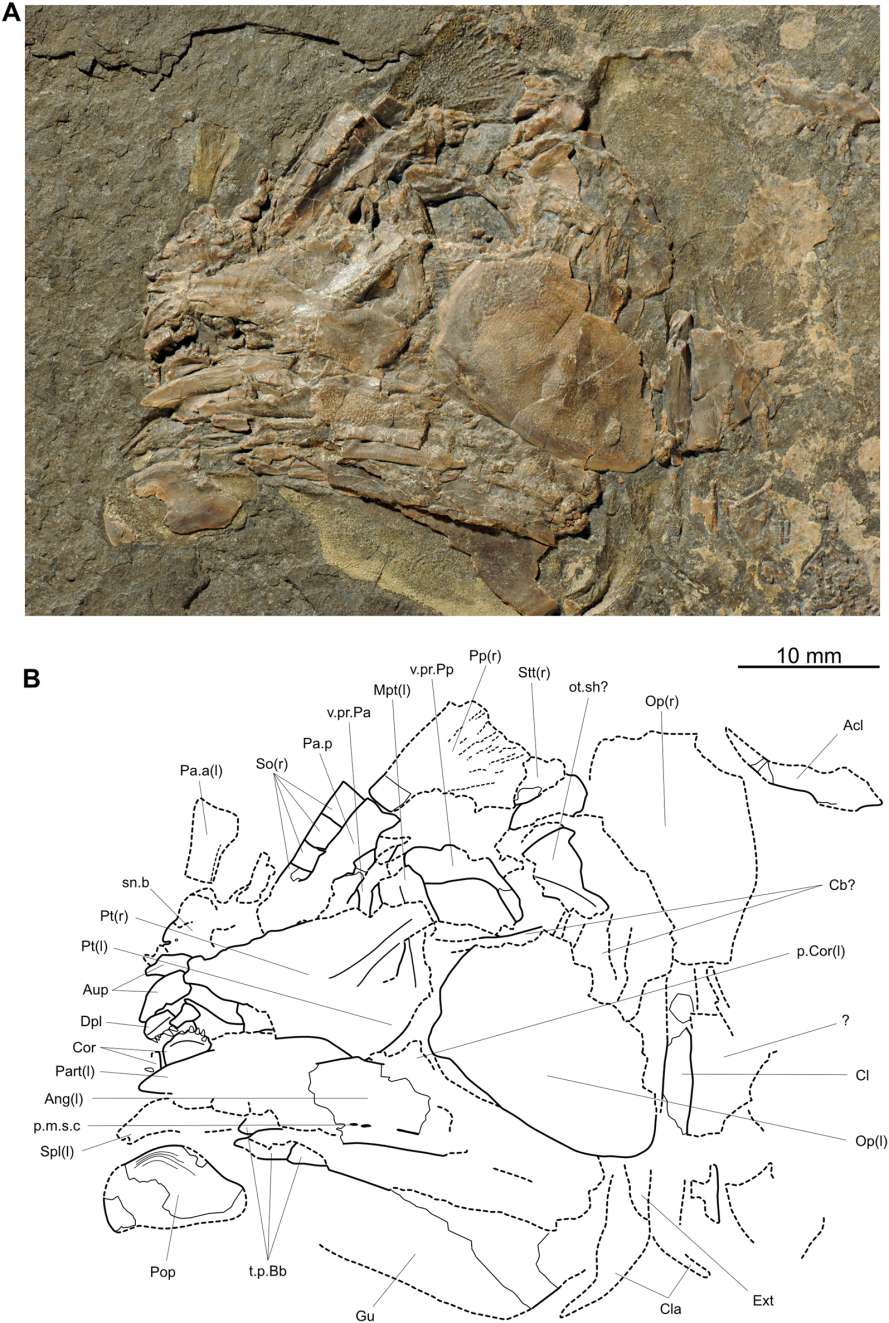
*Ticinepomis peyeri*, holotype (part) PIMUZ T 3925a. **A** Photograph and **B** interpretative drawing of the head in left lateral view

**Fig. 5:**
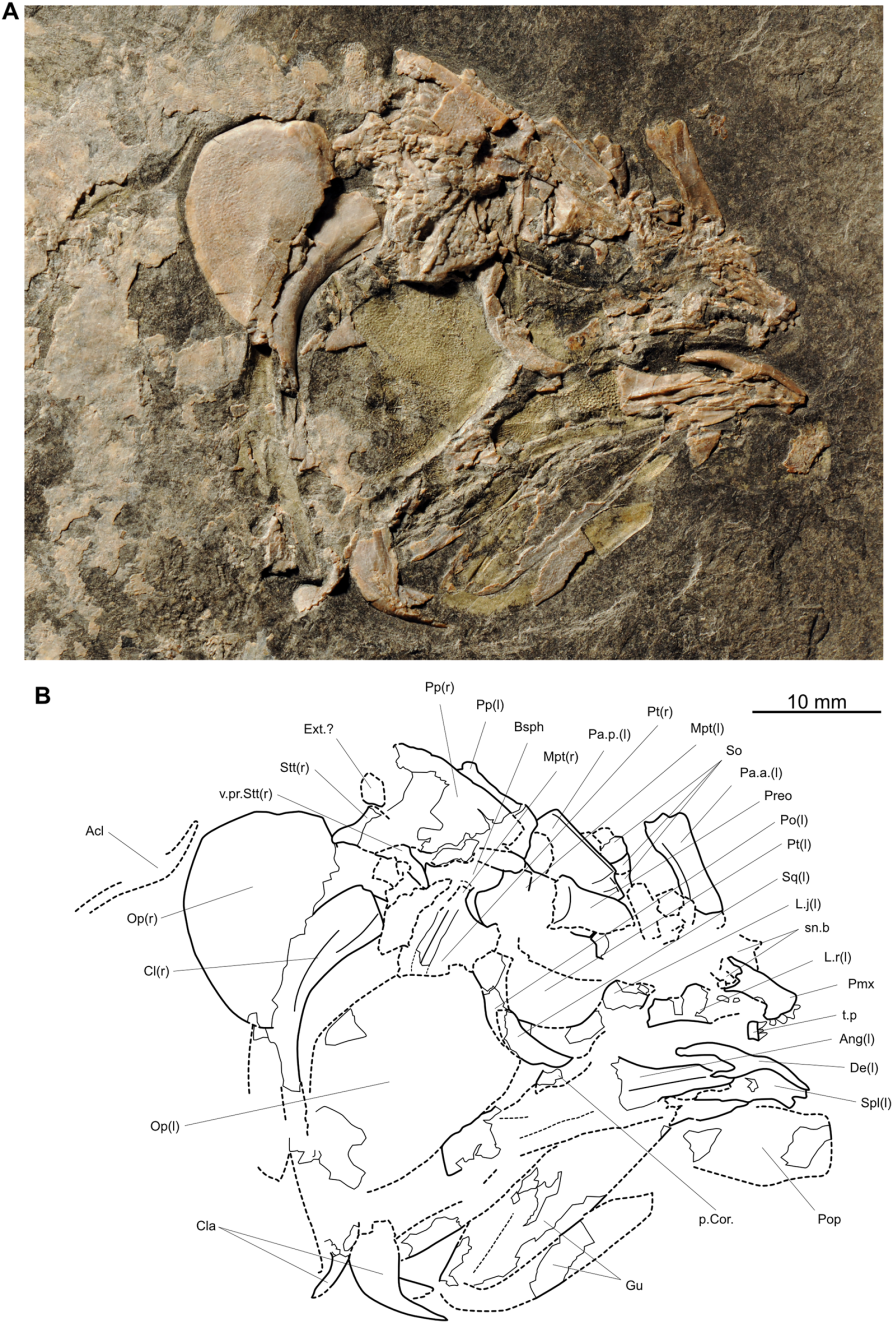
*Ticinepomis peyeri*, holotype (counterpart) PIMUZ T 3925b. **A** Photograph and **B** interpretative drawing of the head in right lateral view

*Ticinepomis peyeri*, a marine species, was described from Monte San Giorgio (Canton Ticino, Switzerland) by Rieppel (1980) and scored by Forey (1998) for the first time in a phylogenetic analysis. Cavin et al. (2013) proposed some modifications of the scoring based on a re-examination of the holotype of *Ticinepomis peyeri* (PIMUZ T 3925) and on specimen PIMUZ A/I 2985 (herein designated the holotype of *Ticinepomis ducanensis*). The re-examination of the holotype of *Ticinepomis peyeri* (PIMUZ T 3925) and the study of new specimens referred herein to *T. peyeri* (PIMUZ T 978, 1513, 2653, 2651) allow a better understanding of some characteristics of this species. It is worth noting that the holotype (PIMUZ T 3925) is difficult to interpret due to the particular separation of the part (PIMUZ T 3925a; Fig. 4) and counterpart (PIMUZ T 3925b; Fig. 5), which passed along a parasagittal plane through the specimen breaking several bones of the skull. The skull was also strongly compressed laterally during fossilization. Therefore, we analysed the holotype by superimposing the photos of the part and counterpart and then varying the transparency of the photos.

Based on the fusion between the supratemporal and the postparietal (an interpretation rejected by Cavin et al. (2013) and in this work), Rieppel (1980) considered that the holotype of *T. peyeri* (PIMUZ T 3925) represents a fully grown individual. Cavin et al. (2013) proposed that this specimen may not represent a fully grown individual but nevertheless admitted that it was unlikely to represent a juvenile individual, because the ossification stage of its skeleton is too advanced (i.e., presence of fully ossified basal plates). This specimen does not indeed represent a juvenile individual but may represent an almost fully grown individual because of its advanced ossification stage and developed ornamentation of the dermal bones.

### Dermal bones of the skull

**Fig. 6:**
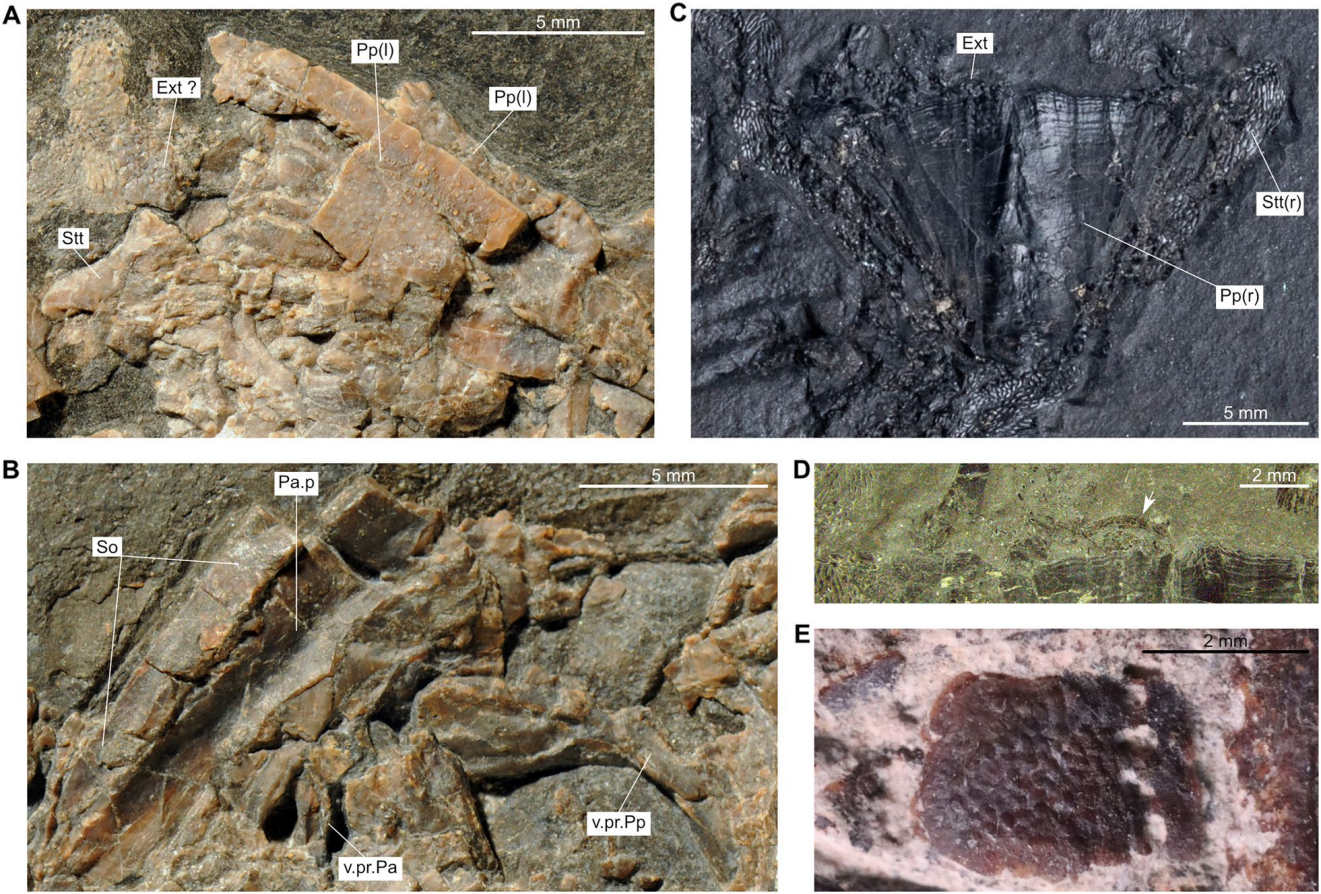
Skull bones of *Ticinepomis peyeri*. **A** Holotype (counterpart) PIMUZ T 3925b, postparietal shield including the right and left postparietals, right supratemporal, and a possible extrascapular. **B** Holotype (part) PIMUZ T 3925a, parietonasal shield including the right posterior parietal, supraorbital series, the descending process of the posterior parietal and the descending process of the postparietal. **C** Specimen (part) PIMUZ T 2651a, postparietal shield with an extrascapular in internal view. **D** Enlargement of C showing the posterior margin of the skull roof with an extrascapular (white arrow), note on the postparietals the growth lines of ossification (about 6 lines) developing parallel to the posterior margin of the postparietals; **E** Specimen PIMUZ T 978, isolated supraorbital showing four pores for the supraorbital sensory canal

The postparietal shield of PIMUZ T 3925 is badly crushed, so that its outlines are not clear (Figs 4, 5, 6A). A sub-complete specimen (PIMUZ T 2651), preserved as part and counterpart, shows a better preserved postparietal shield in internal view (Fig. 6C). The postparietal shield is found detached from the parietonasal shield, and the anterior border forming the joint margin is straight without any trace of overlap areas. Therefore, the parietonasal and postparietal shields are free from each other.

### Parietonasal shield

The snout is poorly preserved in PIMUZ T 3925. Only the premaxilla **(Pmx)** can be distinguished. The posterior snout bones **(sn.b)** are so badly crushed that almost nothing can be described, except the presence of a small round pore followed by a small notch for the sensory canal that is on the only well-delimited margin (anteriorly located) of one of the snout bones (PIMUZ T 3925a; Fig. 4). The premaxilla bears four stout conical teeth (Fig. 5). It is worth noting that the premaxilla is a longitudinally elongated bone (Cavin et al., 2013 fig. 3b; Rieppel, 1980, fig. 2). Such an elongated premaxilla is unusual in coelacanths, but there is no visible suture or limit that would indicate that this ossification corresponds to the fusion of a small premaxilla with the rostral ossicles. Nevertheless, we consider that the overall snout is too poorly preserved to identify its structure with certainty. Therefore, we refrain from identifying this large bone as a rostropremaxilla bone, which is present in some coelacanths such as *Macropoma lewesiensis* (Forey, 1998, fig. 3.19A) for instance.

On PIMUZ T 3925, Rieppel (1980, figs 2, 3) identified a series of poorly preserved bones as the supraorbital series **(So)**, but their preservation precludes further description (Figs 4, 5, 6B). An isolated but well-preserved supraorbital (Fig. 6E) is present in the specimen PIMUZ T 978. This squarish bone, ornamented with coarse roundish tubercles, is perforated by four well-marked round pores. Thus, in *T. peyeri*, the supraorbital sensory canal opens through many pores in the lateral series similarly to *Whiteia woodwardi* (Forey, 1998). This situation contrasts with *Foreyia* where this canal opens in a continuous groove (Cavin et al., 2017) or with *Diplurus newarki* where it opens with a few pores along the sutural contact of bones (Schaeffer, 1952).

Rieppel (1980, figs 2, 3) identified on the holotype (PIMUZ T 3925b) and figured in his reconstruction a lateral rostral **(L.r)**. Although he correctly identified the bone, he reconstructed it as being very short, which is an odd situation for a coelacanth. The anterior part of the bone is strongly crushed, but the counterpart (Fig. 5) suggests a longer bone than the reconstructed one, with the shape reminiscent of the lateral rostral of most other coelacanths. The ventral process of the lateral rostral does not appear to be developed at all.

**Fig. 7:**
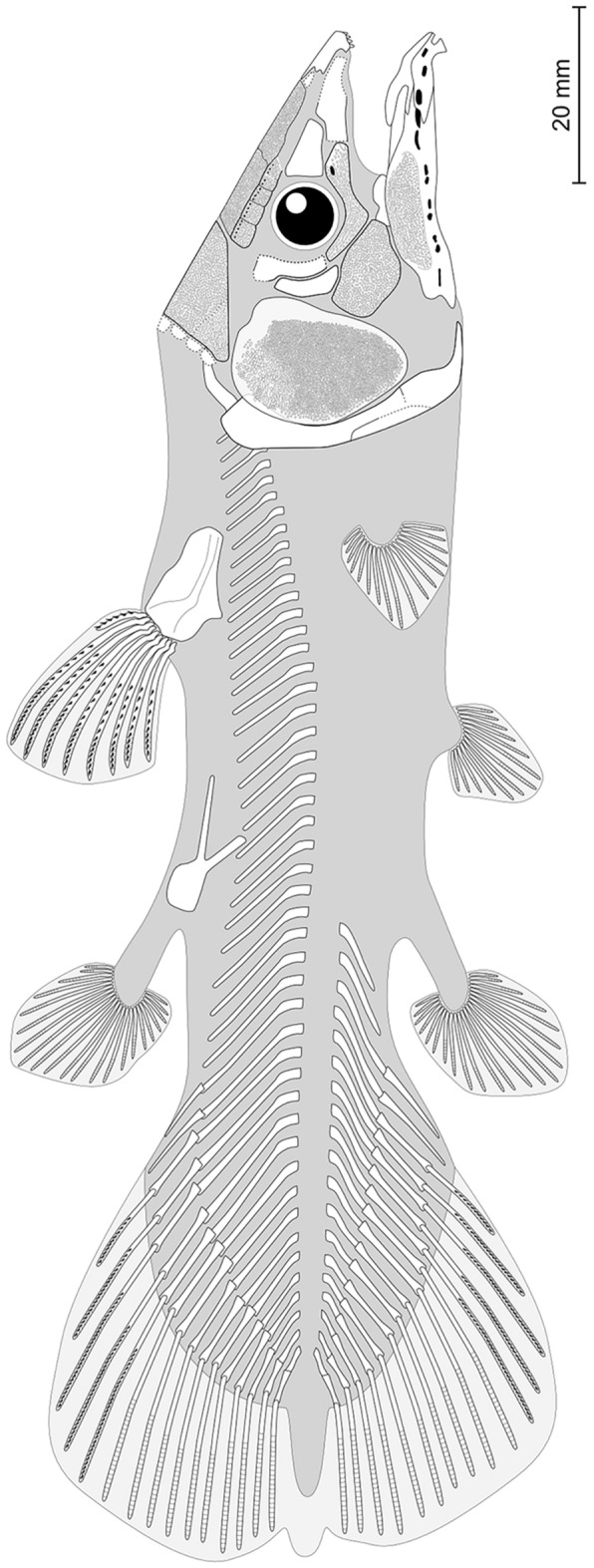
Tentative reconstruction of *Ticinepomis peyeri*. Based on the holotype (PIMUZ T 3925) and other referred specimens (PIMUZ T 978 and 2651)

On PIMUZ T 3925b above the anterior dorsal margin of the lachrymojugal, lies a triangular to ovoid bony plate that is interpreted here as a large preorbital **(Preo)** (Fig. 5). The bone appears to be visible in internal aspect, which makes impossible to determine if it is ornamented. Although crushed, the interpreted posterior margin of the bone is curved in such a way that it would thus correspond to the orbital margin. There are no openings or visible notches for the posterior openings of the rostral organ on the ventral margin that is crushed. The reconstruction of the cheek of *T. peyeri* (Fig. 7) and the posterodorsal curvature of the lateral rostral agrees with the presence of a preorbital. It is worth noting that Rieppel (1980, fig. 2) tentatively identified the bone interpreted here as the preorbital as the basisphenoid. However, there is no structure (as, e.g., antotic process) on this bone supporting this identification.

On PIMUZ T 3925a, Rieppel (1980, fig. 1) identified under the posterior parietal (his frontal) a bony element as the right metapterygoid. However, this element is reminiscent of a ventral process of the skull roof in comparison with other taxa such as in *Macropoma* (Forey, 1998, fig. 6.10A). According to its position below the posterior parietal **(Pa)**, it is interpreted here as the descending process of the posterior parietal **(v.pr.Pa)** (Figs 4, 6B). It is worth noting that Cavin et al., (2013, fig. 3a) previously identified a descending process of the posterior parietal, but on the basis of another poorly preserved bony element.

### Postparietal shield

On PIMUZ T 3925a, Rieppel (1980, fig. 1) identified a curved bone as the postorbital, because it resembles the left postorbital identified on the counterpart. However, its thin ventral region and its position directly below the right postparietal bone suggest rather that this bone is the descending process of the postparietal **(v.pr.Pp)** (Figs 4, 6B). This bony element, tapering ventrally, has a large dorsal portion that represents the point of attachment with the postparietal bone.

Below the right supratemporal **(Stt)**, Cavin et al., (2013, fig. 3b) labelled an element on the counterpart as a possible posterior wing of the prootic. Regarding its position and relationship with the supratemporal (PIMUZ T 3925b), this element, similar to the descending process of the parietal identified above, is better interpreted as the descending process of the supratemporal **(v.pr.Stt)** (Figs 5, 6A).

Based on the reconstruction by Rieppel (1980, fig. 3), Forey (1998) scored the posterior margin of the skull roof as being embayed while Cavin et al. (2013) corrected this feature as straight. In PIMUZ T 3925b, the posterior margin of the postparietals is completely crushed precluding to define its exact outline (Figs 5, 6A). The supratemporal, which is somewhat better preserved, appears to have almost straight posterior and lateral margins. In the specimen PIMUZ T 2651 (Fig. 6C, D), the posterior margin of the postparietals appears to be clearly straight along all its width.

On PIMUZ T 3925b, posterior to the right postparietal is a crushed bony element that may correspond to a small extrascapular **(Ext.?)** (Fig. 5). This interpretation is reinforced by the presence on the specimen PIMUZ T 2651 of a small and well-preserved extrascapular lying behind the postparietal (Fig. 6C, D). Other extrascapular bones are missing in this specimen, indicating that these bones were likely free from the skull. Although it is currently impossible to assess the exact number of extrascapulars, there were probably more than one extrascapular forming the posterior margin of the skull roof.

The imprint of the postparietal on PIMUZ T 3925a shows short and straight to undulating marks oriented anteroposteriorly extending from the posterior margin to almost the middle portion of the bone (Fig. 4). These marks are reminiscent of the grooves for the anterior branches of the supratemporal commissure that are known only in some Latimeriidae, such as *Macropoma* (Forey, 1998, fig. 3.21) for instance. As it is preserved as an imprint, we refrain from identifying this structure as the anterior branches of the supratemporal commissure.

### Dermal bones of the cheek

In all specimens of *Ticinepomis*, the bones of the cheek are very difficult to interpret due to the poor preservation of this region of the skull.

On PIMUZ T 3925b, Rieppel (1980, fig. 2) identified a tubular bone as the postorbital **(Po)** lying directly in contact with a comma-shaped squamosal **(Sq)**. It appears, however, that these bones are not in natural contact with each other but rest one above the other (Fig. 5) (the squamosal hides the ventral portion of the postorbital). Therefore, the postorbital is in fact longer than in the restoration provided by Rieppel (1980, fig. 3). The postorbital and the squamosal are two bones reduced to narrow tubes surrounding the sensory canal. The visible postorbital and squamosal belong to the left side of the skull and are preserved in mesial aspect, thus not allowing to see their ornamentation. However, it should be noted that on the right pterygoid, there are traces of ornamentation composed of coarse tubercles that could belong to the right postorbital. Moreover, on PIMUZ T 2651 is a heavily ornamented tubular bone that may be a postorbital, but this part of the fossil requires more preparation to confirm this identification. Although Rieppel (1980, fig. 3) reconstructed the postorbital spanning the intracranial joint, our reconstruction (Fig. 7) suggests rather that the postorbital lies well behind the intracranial joint.

No independent jugal is identified and this bone was likely absent.

In PIMUZ T 3925, Rieppel (1980, fig. 1) identified a large bone as the preopercle **(Pop)** (Figs 4, 5). We agree with his identification. Compared to other bones of the cheek, this undifferentiated preopercle is proportionally large. Our reconstruction (Fig. 7) indicates that the preopercle is positioned under the squamosal and postorbital rather than posteriorly to them. Although this bone is preserved in mesial view, some broken portions clearly indicate that the bone was ornamented with coarse tubercles.

**Fig. 8:**
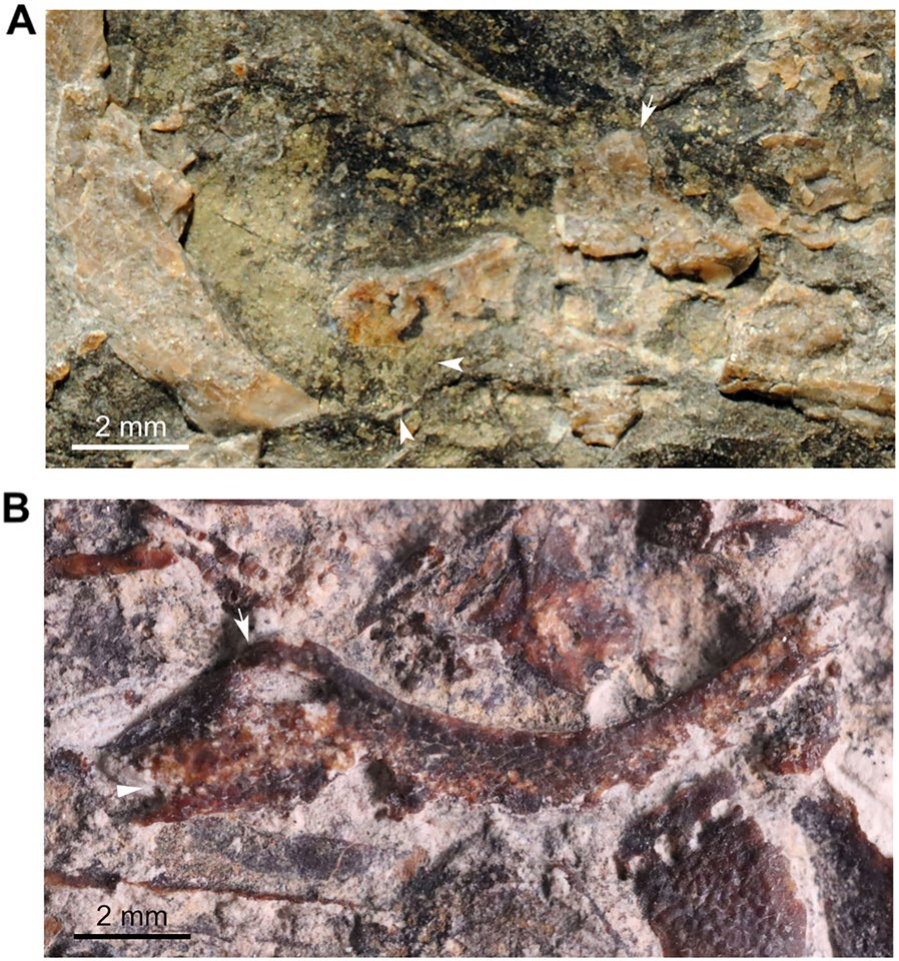
Lachrymojugal of *Ticinepomis peyeri*. **A** Holotype PIMUZ T 3925b, lachrymojugal showing the posterior portion with the ventral thickening (white arrowhead) preserved as an imprint and the anterior portion preserved as crushed bones with the anterior dorsal angle (white arrow indicates where the bone starts to angle); see Fig. 5 for a detailed explanation. **B** Specimen PIMUZ T 978, lachrymojugal showing the anterior dorsal angle (white arrow indicates where the bone starts to angle) above the large pore for the infraorbital sensory canal and the location of the groove for the posterior nasal tube (white arrowhead)

Based on a poorly preserved imprint in PIMUZ T 3925b, lying anteriorly to the preopercle and the squamosal, Rieppel (1980, fig. 3) reconstructed the lachrymojugal **(L.j)** as a narrow and strongly ventrally curved tube. Following Rieppel (1980) and Cavin et al. (2013), we agree that the lachrymojugal (Figs 5, 8A) is a bone with a peculiar shape having a curved and thicker triangular ventral margin. This characteristic is similar, but less developed, than on the lachrymojugal of *Foreyia* (Cavin et al., 2017, fig. S6, their lachrymojugal + squamosal). The interpretative drawing of Rieppel (1980, figs 2, 6) suggests that the anterior portion of the lachrymojugal is expanded or angled, but the poor preservation of this bone in PIMUZ T 3925 does not allow to confirm this assumption. On PIMUZ T 978, there is a better preserved lachrymojugal (Fig. 8B). The shape of the bone fits perfectly with the poorly preserved lachrymojugal of PIMUZ T 3925b as drawn by Rieppel (1980, fig. 2). Unfortunately, the ventral outline of the bone is crushed and the posterior portion is sunk in the matrix, and is covered by a supraorbital and another bone. The anterior end of the lachrymojugal forms a small angle, as in *Whiteia woodwardi* (Forey, 1998, figs 4.14 and 4.15) for instance. The surface of the lachrymojugal is covered with a wavy elongated ornamentation forming ovoid tubercles, which makes it difficult to see the pores for the sensory canal. In the middle of the bone are some very tiny roundish structures, difficult to observe, that may be pores, but this identification is uncertain. On the anterodorsal margin is a large ovoid pore similar to that observed on the lachrymojugal of *Whiteia woodwardi* (Forey, 1998, figs 4.14 and 4.15). It is assumed here that the infraorbital sensory canal, at least, opens through a few large pores. A notch is dug in the anterior ventral corner that is interpreted here as the mark for the posterior nasal tube (Fig. 8B). This identification fits with the pattern of the infraorbital sensory canal that opens, and then passes, above this notch, as for instance in *Macropoma* or *Latimeria* (Forey, 1998).

### Lower jaw

**Fig. 9:**
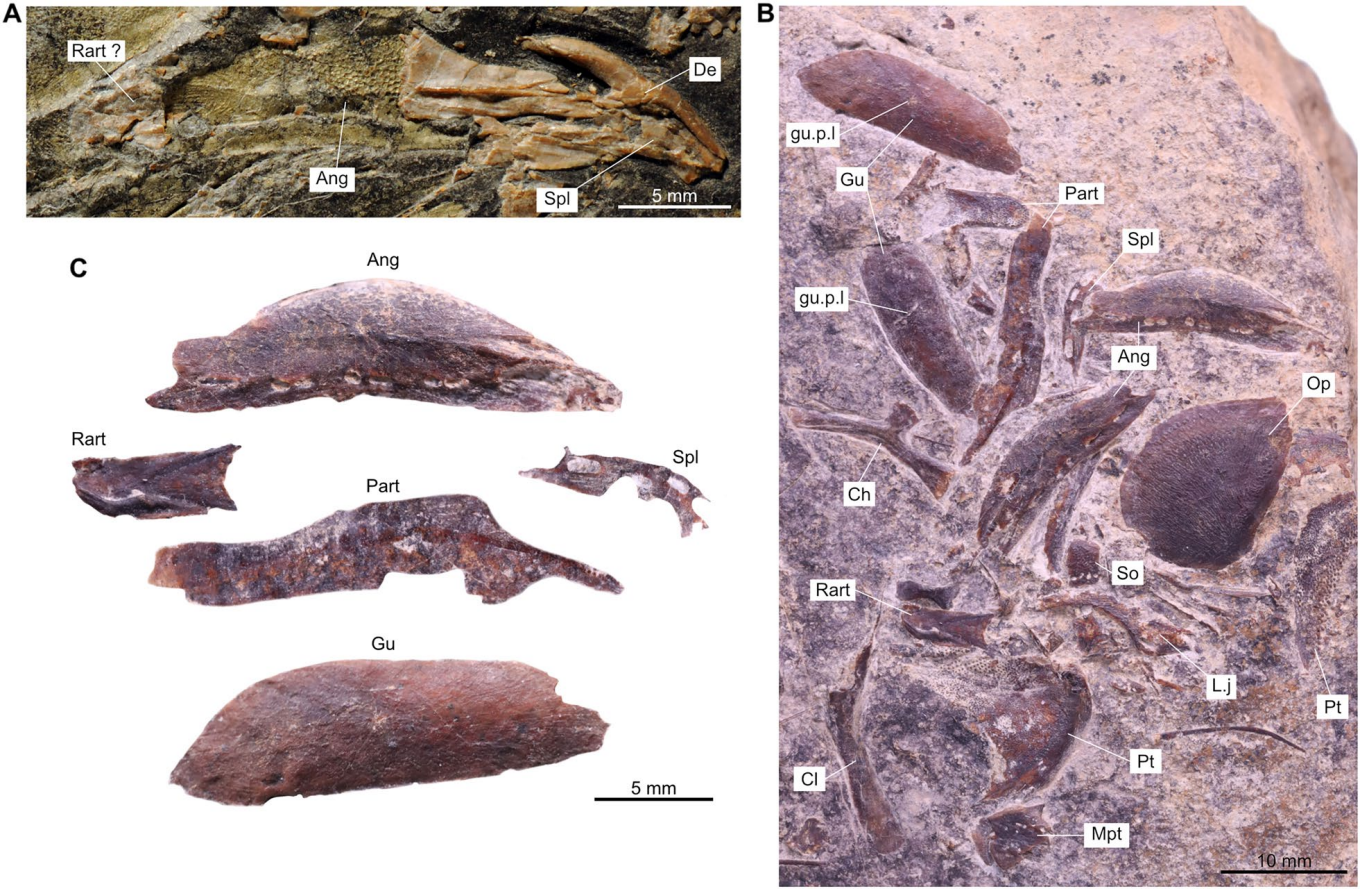
Lower jaw of *Ticinepomis peyeri*. **A** Holotype PIMUZ T 3925b, left lower jaw showing the dentary, the splenial, the angular, and possibly the retroarticular. **B** Specimen PIMUZ T 978, scattered bones of the lower jaw including a retroarticular, angulars, a splenial, prearticulars, and gular plates. **C** Bones of the lower jaw of **B** pulled apart and arranged in accordance with bones of the lower jaw of the holotype PIMUZ T 3925b (note that the gular pit line on this gular plate is less visible than on the other gular plate visible in **B**)

The lower jaw of the holotype of *T. peyeri* (PIMUZ T 3925) is much better preserved in its anterior part than in its posterior part (Figs 4, 5 and 9A). On PIMUZ T 978, we identified a bone as an isolated retroarticular (Fig. 9B, C). It is rectangular with a rounded posterior margin and bears a long surface that is interpreted as the facet of articulation for the quadrate.

On PIMUZ T 978, the bones of the lower jaw, except the dentary, are well visible (Fig. 9B, C). The angular **(Ang)** is a shallow and approximately parallel-sided bone (Fig. 9B, sC). There is a total of eight pores for the mandibular sensory canal **(p.m.s.c)** on its ventral margin. The most posterior and anterior pores are elongated and ovoid, while the six other pores in the middle area are almost round and open as three closely spaced pairs. There is no visible oral pit line, but the ornamentation makes difficult to ascertain the absence of this feature.

On PIMUZ T 3925a, the prearticular **(Part)** is difficult to distinguish because of the mode of preservation. On PIMUZ T 978, two prearticulars are well preserved, one entirely in lateral view and one partially in mesial view (Fig. 9B, C). It is a shallow bone with parallel-sided margins that tapers anteriorly. On the lateral side of this bone runs a long ridge, well marked from the middle to the anterior margin, along the mid-depth of the bone. On the partially preserved prearticular, the surface is densely covered with small conical round teeth. Few teeth are ornamented with very fine and faint striae that are hard to detect.

The splenial **(Spl)** appears to be angled downwards anteriorly on PIMUZ T 3925b (Figs 3C, 5, 9A), a peculiar feature that is even more pronounced on the splenial of PIMUZ T 978 (Fig. 9B, C). In this specimen, the splenial is well preserved and displays additional characteristics not visible on PIMUZ T 3925. The bone is smooth and unornamented like the dentary but unlike the angular. The mandibular sensory canal opens laterally in the mid-depth of the splenial with three large and elongated rectangular pores. There is one large and elongated pore that opens between the splenial and the angular. On the anterior margin of the splenial is a large notch, which, when in contact with its antimere, forms a large symphysial pore. A symphysial pore on the splenial is also present in *Foreyia* and *Whiteia woodwardi* (drawn but not labelled or described in Forey, 1998, fig. 5.9A), and possibly in *Luopingcoelacanthus* (not described but suggested by the illustrations provided by Wen et al., 2013, figs 1, 2A). The shape of the splenial and the arrangement of the pores of *T. peyeri* are reminiscent of the pattern in *Foreyia*, except that in the latter, there are only two pores in the mid-height of the bone (Cavin et al., 2017, fig. S6) and not three pores like in *T. peyeri*.

The dentary **(Den)** of *T. peyeri* bears a strong and well-developed hook-shaped process (Figs 5, 9A). The presence of a dentary pore in *T. peyeri* remains unknown because of the poor preservation in available specimens.

**Fig. 10:**
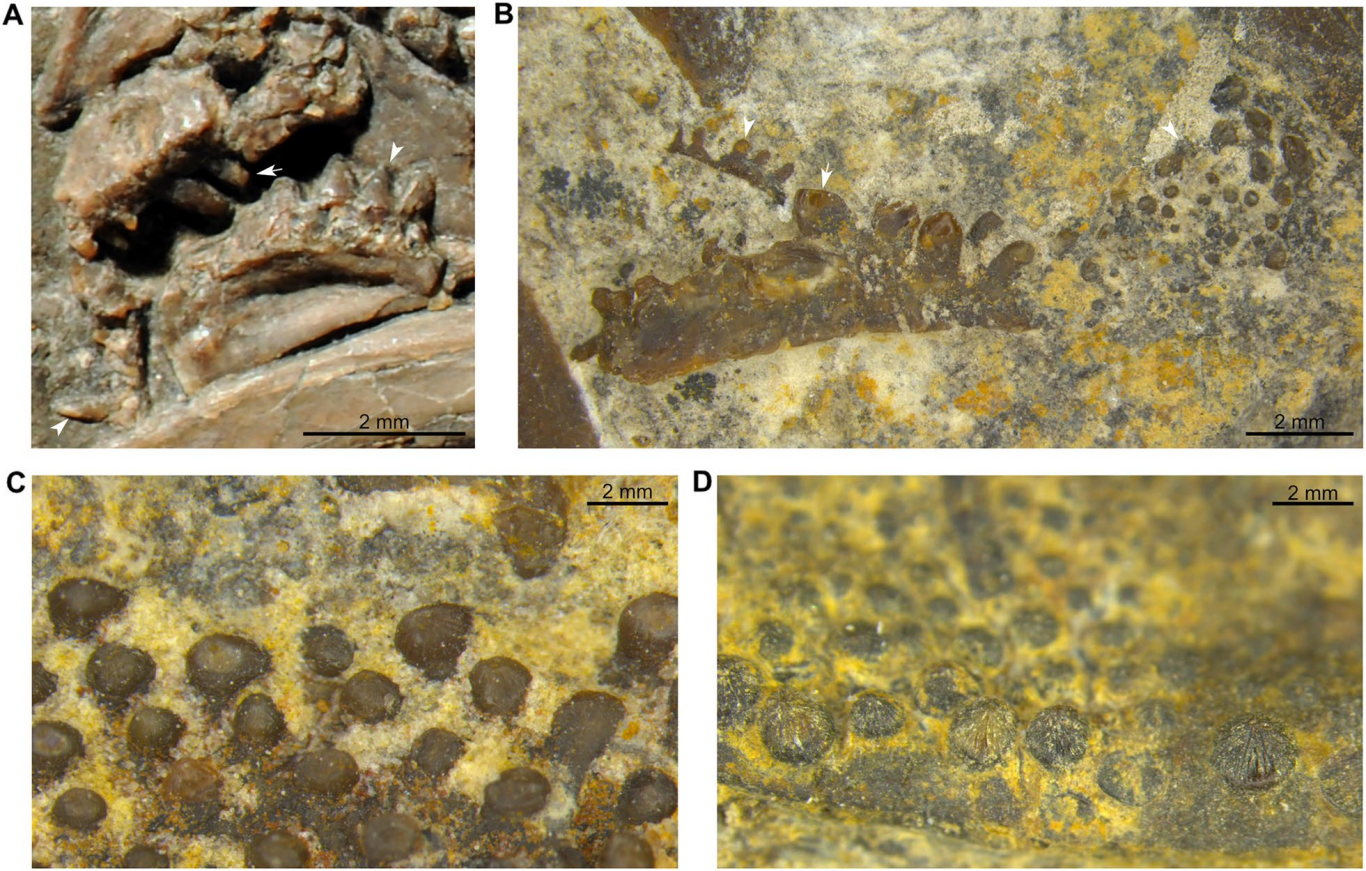
Dentition of *Ticinepomis peyeri*. **A** Holotype (part) PIMUZ T 3925a, coronoid (white arrow-head) and dermopalatine (white arrow). **B** PIMUZ T 978, coronoid or dermopalatine (white arrow), tooth plates (white arrow-head) and **C** teeth of the pterygoid. **D** PIMUZ T 1513, teeth of the parasphenoid along its external portion

Rieppel (1980) reported on PIMUZ T 3925a the presence of two coronoids (the ‘precoronoids’ of Rieppel), just above the location of the dentary based on PIMUZ T 3925b. At least five conical teeth are borne on the best-preserved coronoid **(Cor)** (Figs 4, 10A). On PIMUZ T 3925b is a small bony plate with three visible conical teeth, which is regarded as a tooth plate **(t.p)** (Fig. 5), because it is smaller and has a different shape from the coronoids. On PIMUZ T 978, there are some similar small bony structures with teeth that may also be interpreted as coronoids and tooth plates (Fig. 10B).

The bone on PIMUZ T 3925b labelled by Rieppel (1980, fig. 2) as a coronoid is more precisely the principal coronoid **(p.Cor)** preserved as a very fragmented bone on a poor imprint (Figs 4, 5). Although poorly preserved, this bone is not sutured to the angular. It is worth noting that Forey (1998) scored in his phylogenetic analysis the principal coronoid in a reverse way, i.e., as sutured to the angular.

The gular plates **(Gu)** are poorly preserved but clearly discernible on PIMUZ T 3925 (Figs 4, 5). On PIMUZ T 978, two well-preserved smooth, without any trace of tubercular ornamentation gular plates are preserved (Fig. 9B, C). Both bear small well-marked gular pit line **(gu.p.l)** in their middle portion. On the gular plate of PIMUZ T 3925b (Fig. 5) is the imprint of a ridge running parallel to the lateral edge along the anteroposterior axis, which is regarded as the ridge observed in *Megalocoelacanthus* and *Latimeria* (Dutel et al., 2012) for instance, corresponding to the insertion point of the anterior and posterior ramus of the intermandibular muscle.

### Neurocranium, palatoquadrate, parasphenoid, and gill arches

**Fig. 11:**
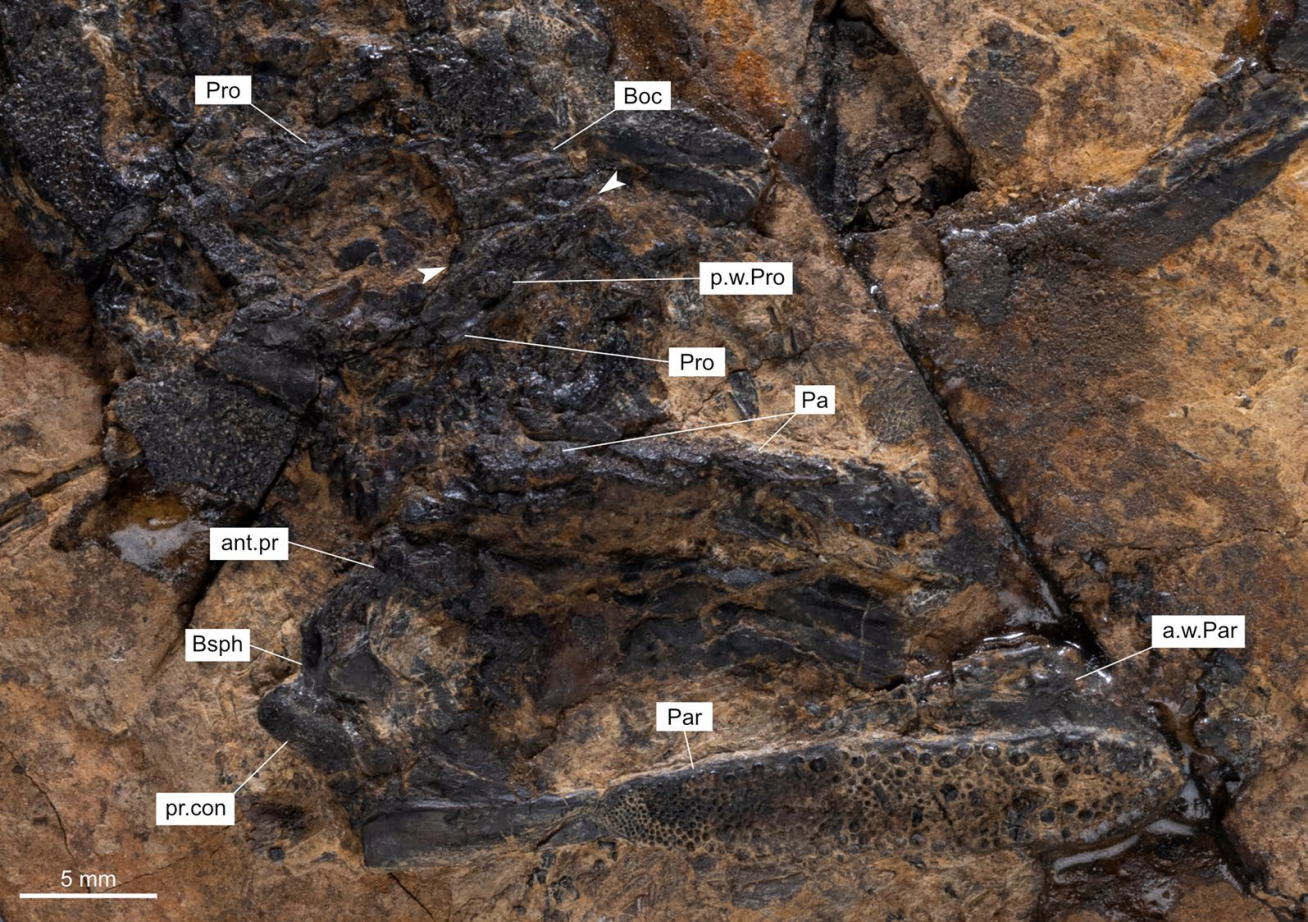
Neurocranium of *Ticinepomis peyeri* (PIMUZ T 1513). The suture between the basioccipital and the prootic is indicated by white arrows

On PIMUZ T 1513, bones of the neurocranium are well preserved in natural position under the parietals (Fig. 11) and most of the following characters are described from this specimen. The neurocranium appears to be derived among coelacanths by having the orbitosphenoid and basisphenoid regions separate from one another, the temporal region not lined with bone and the otico-occipital separated to prootic/opisthotic. On the basisphenoid **(Bsph)** (Fig. 11), the paired processus connectens **(pr.con)** are positioned in such a way that they do not meet the parasphenoid. No basipterygoid processes are present on the basisphenoid. The antotic process **(ant.pt)** is unfortunately broken, but its emplacement can be detected.

The basioccipital **(Boc)** is ossified as an independent bone (Fig. 11), which is a feature also found in *Ticinepomis ducanensis* sp. nov. (Cavin et al., 2013, fig. 5). The prootic **(Pro)** is attached to the basioccipital by a wavy complex suture (Fig. 11). The prootic develops a small posterior wing **(p.w.Pro),** as it can be observed in *Macropoma* (Forey, 1998, fig. 6.10B) for instance. The otico-occipital portion of the neurocranium of *Ticinepomis* then appears to be ossified into distinct units, which is a characteristic found in derived coelacanths (Forey, 1998).

The parasphenoid **(Par)** is preserved in ventral view (Fig. 11). The toothed portion is well developed and extends on the two-thirds of the overall ventral surface of the parasphenoid. On this surface are borne conical and pointed teeth with fine striae (Fig. 10D). Along the external portion of the mid-length of the parasphenoid toothed patch, the teeth are large and decrease in size posteriorly until they are reduced to tiny bulges on the most posterior part. The teeth are small and of equal size across the median portion of the bone. The parasphenoid is not pierced by the foramen for the buccohypophysial canal, meaning that this canal is closed. Anteriorly, the dorsal margin of the parasphenoid bears a well-developed ascending lamina, which lies in contact with a separate lateral ethmoid.

**Fig. 12:**
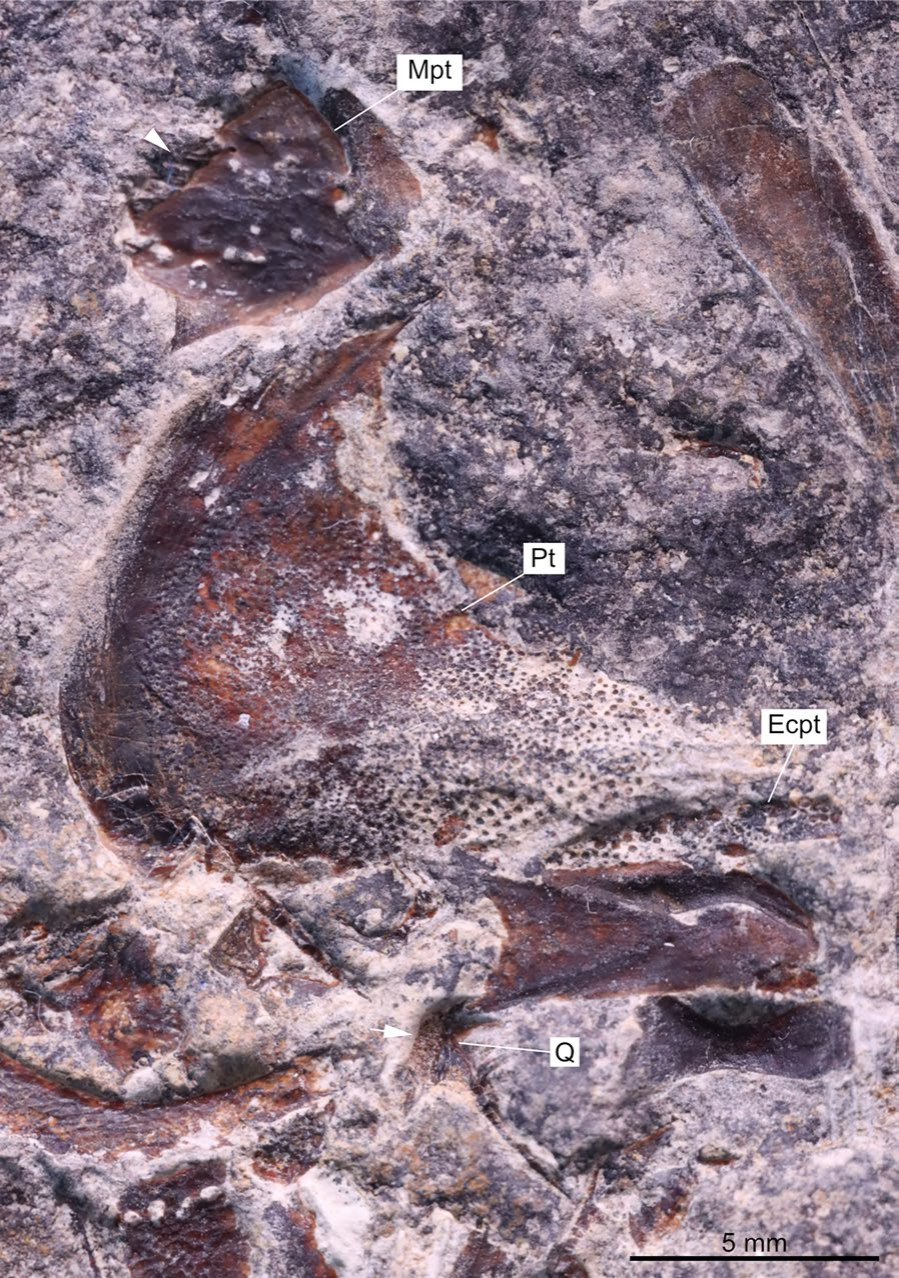
Palatoquadrate of *Ticinepomis peyeri* (PIMUZ T 978). The bones of the palatoquadrate, which include the metapterygoid, pterygoid, quadrate, and ectopterygoid, are detached from each other; white arrowhead on the metapterygoid indicates the surface of articulation with the dorsal part of the pterygoid; white arrow indicates the condyle of the quadrate. Scale bar: 5 mm

In PIMUZ T 3925, Rieppel (1980, fig. 1) already identified bones of the palatoquadrate, but the quadrate is unknown and the shape of the metapterygoid and pterygoid are hard to detect because of the peculiar preservation of the specimen. Only two roughly triangular autopalatine **(Aup)** and a bony element with strong conical teeth interpreted as the dermopalatine **(Dpl)** (palatinum of Rieppel, 1980) can be clearly discerned (Figs 4–5). In PIMUZ T 978, all the bones of the palatoquadrate (Fig. 12), including a metapterygoid **(Mpt)**, a pterygoid **(Pt)**, a quadrate **(Q)**, an ectopterygoid **(Ecpt)**, and a possible dermopalatine (could also be a coronoid), are well preserved. All these bones, except the possible dermopalatine, are preserved close to but detached from each other. The metapterygoid is a robust and almost square-shaped bone, while in PIMUZ T 3925a and in PIMUZ T 2653, the metapterygoid appears to be narrower. The mesial surface of the pterygoid of PIMUZ T 978 is densely covered with small-striated conical teeth (Fig. 10C). Unfortunately, the ventral margin of the pterygoid is not well preserved and we cannot assess the condition of the ventral swelling. The ectopterygoid is elongated and bears small conical teeth that are apparently smooth. The isolated bone of PIMUZ T 978, identified either as a dermopalatine or a coronoid (Fig. 10B), bears strong conical teeth, which seem to be smooth with no detectable striae, unlike the teeth observed on the pterygoid and parasphenoid.

Three basibranchial tooth plates **(Bb.t.p)** are visible in PIMUZ T 3925 (Rieppel, 1980, fig. 1) and include two anterior and one posterior basibranchial tooth plates (Fig. 4). The two anterior basibranchial tooth plates are paired and not fused together. Unfortunately, the posterior portion of the basibranchial tooth plate’s series is covered by bones of the jaw, making it impossible to determine if there are two or more pairs of basibranchial tooth plates. The teeth of the basibranchial tooth plates cannot be seen, because the bones are preserved in ventral aspect.

In PIMUZ T 978, there is a well-preserved ceratohyal **(Ch)** (Fig. 9B). This long and curved bone has an expanded distal extremity and a thinner proximal extremity. At its mid-length is a well-developed ventral process, reminiscent in shape to the ventral process of the ceratohyal of *Luopingcoelacanthus* (Wen et al., 2013, fig. 3E).

### Axial skeleton

In his description of PIMUZ T 3925, Rieppel (1980) mentioned 51 neural arches, including 33 and 18 neural arches in the abdominal and the caudal regions, respectively. However, regarding the interpretative drawing of Rieppel (1980, fig. 4), only 47 arches are clearly drawn, including 32 neural arches, 21 haemal arches, and 15 radials. In the upper lobe of the caudal fin, we counted directly on the specimen 15 radials supporting each a fin ray, plus two anterior supplementary radials supporting no fin ray. In the lower lobe, we count 14 to 15 radials supporting each a fin ray plus two anterior supplementary radials supporting no fin ray. PIMUZ T 2651 displays the axial skeleton including neural and haemal arches in natural position, except for the five anterior first neural arches slightly detached and away from the rest of the column. In this specimen, we count a total of 47 neural arches and about 19 haemal arches. Unfortunately, the caudal fin rays, radials, and haemal arches are difficult to count, because they are preserved compressed against one another. In the upper lobe, we counted 15 rays plus one posterior most ray that seems to belong to the supplementary lobe, not being supported by a radial. Anteriorly, there is at least one radial, possibly two, that supports no ray. Thus, 15 rays and 15–17 radials in the upper lobe of the caudal fin are recognised. This number of radials and fin rays is consistent with PIMUZ T 3925. The three anterior most rays in both lobes bear small and sharp denticles. *T. peyeri* has 31 or 32 neural arches which are not incorporated in the caudal fin and 15 or 16 neural arches in the caudal fin for a total of 47 neural arches. Posterior neural and haemal arches are not abutting one another as already stated by Forey (1998). The caudal fin is composed of 15 and 14–15 radials each supporting one fin ray plus two additional non-supporting radials in the upper and lower lobes, respectively. Therefore, there is a one-to-one relationship between the radials and the fin rays in the tail, as already stated by previous workers.

### Paired fins

In his emended diagnosis of *Ticinepomis*, Forey (1998) stated that the fin rays are slightly expanded, an assumption rejected here. Indeed, all fins of *T. peyeri* present slender rays and are clearly not expanded as in *Libys polypterus* (Ferrante et al., 2022; Lambers, 1992, fig. 1 and pl. 1) for instance.

### *Pectoral girdle and fins*

**Fig. 13:**
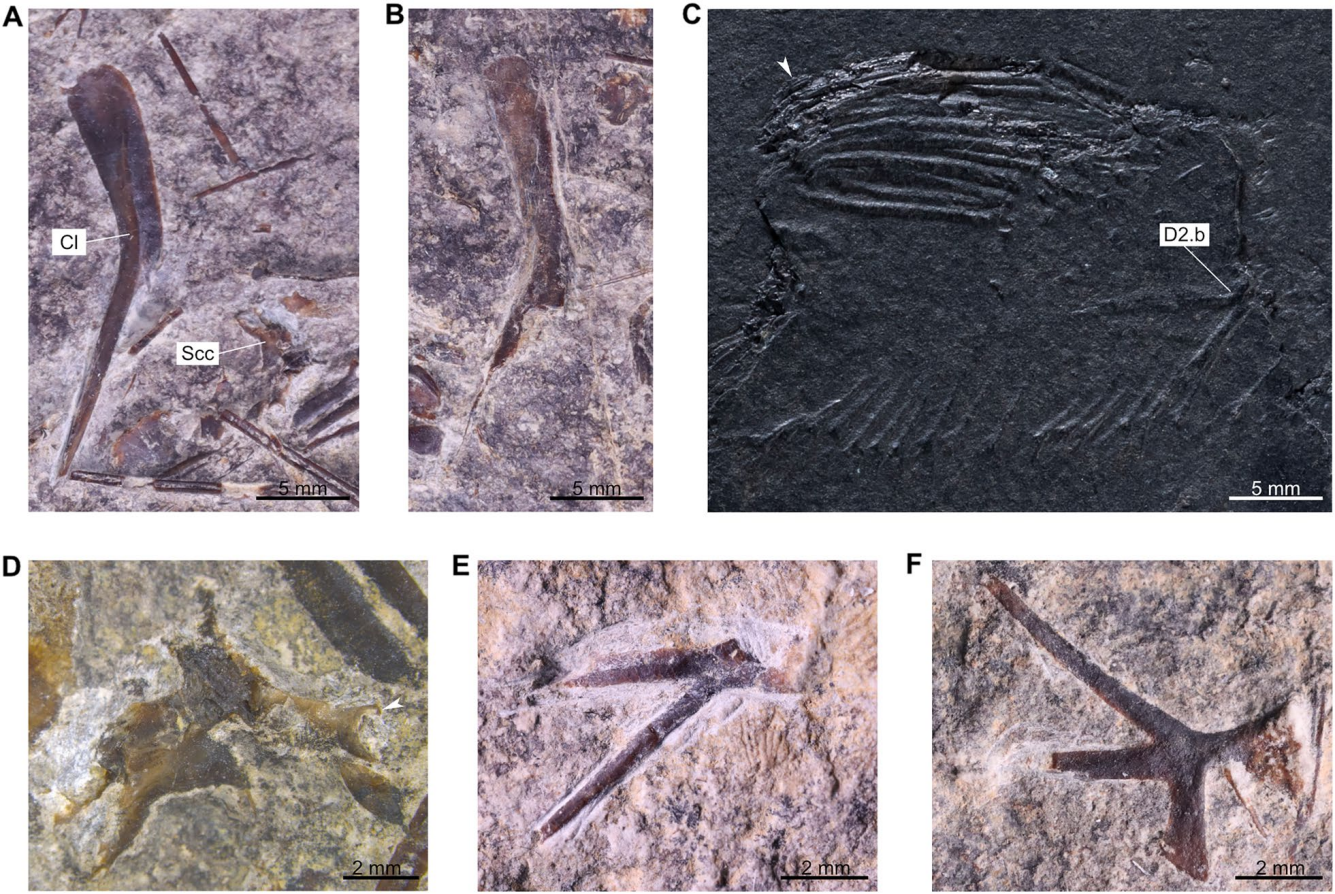
Girdles and fins of *Ticinepomis peyeri*. **A** PIMUZ T 978, left cleithrum in lateral view showing the enlarged dorsal portion and the scapulocoracoid with its articular facet (white arrow). **B** PIMUZ T 978, right cleithrum in mesial view. **C** PIMUZ T 2651b, anterior dorsal fin showing the first smaller fin ray (white arrowhead) and the basal plate of the posterior dorsal fin (white arrow). **D** PIMUZ T 978, enlargement of the scapulocoracoid with its articular facet (white arrow) exposed in (**A**). **E** PIMUZ T 978, basal plate of the posterior dorsal fin or the anal fin. **F** PIMUZ T 978, basal plate of the pelvic fin

On PIMUZ T 978, two cleithra each with enlarged distal portion (Fig. 13A, B), as in PIMUZ T 3925, and one scapulocoracoid with the articular head for the pectoral fin (Fig. 13A and D) are preserved. According to Rieppel (1980), there is an indication of 17 rays in the pectoral fin, a count that we were unable to confirm due to the poor preservation of this fin. On PIMUZ T 3925b, Rieppel (1980, fig. 2) identified a bone as a remnant anocleithrum (his ‘supracleithrum’), which is covered ventrally by the cleithrum and dorsally by sheets of unidentified bony elements. On both parts of PIMUZ T 3925, we identified another fragmentary bone as an anocleithrum **(Acl)** (Fig. 4, 5). Despite its poor state of preservation, the anocleithrum is simple and sigmoid in shape.

### *Pelvic girdle and fin*

On PIMUZ T 978 is a well-preserved basal plate of the pelvic fin (Fig. 13F), which presents posterior, lateral, and medial processes. According to Rieppel (1980), there is an indication of 13 rays in the pelvic fin, but he pointed out that this count may be too low by comparison with other coelacanths. Nevertheless, this low number may also be real. Indeed, a low number of rays in the pelvic fin is known in some coelacanths, such as for instance in the sister genera *Foreyia* and *Hadronector*, which have 12 and 10–12 rays in the pelvic fin, respectively (Cavin et al., 2017; Forey, 1998).

### Unpaired fins

#### Anterior dorsal fin

The basal plate of the anterior dorsal fin is roughly triangular shaped with rounded anterior and posterior margins and a ventral margin with a distinct spine posteriorly directed (Rieppel, 1980). The ventral margin of the bone is smooth and slightly concave.

In the anterior dorsal fin of PIMUZ T 3925, Rieppel (1980) counted eight rays of which some are poorly preserved. In PIMUZ T 2651, we counted eight long rays following one to two considerably smaller anterior ray(s), which appears to be unsegmented (Fig. 13C). A careful look at PIMUZ T 3925 shows that there is at least one additional very small ray placed anteriorly to the longest rays. This small anterior ray is hard to see, because only a very small proximal part is preserved, while the rest is preserved as a very poor imprint. Therefore, the anterior dorsal fin of *T. peyeri* is composed of a total of 9–10 rays, including eight long, segmented rays preceded by 1–2 smaller, unsegmented rays.

Such an arrangement of fin rays in the anterior dorsal fin, showing a few smaller anterior rays preceding considerably longer rays, is, to our knowledge, unique to a few coelacanths. This condition is extreme in the anterior dorsal fin of *Allenypterus* (Forey, 1998, fig. 11.2). It is also observed in a few other coelacanths but in a less extreme way than in *Allenypterus*. In those forms, only the first and/or second anterior rays are smaller than the posterior ones. The anterior rays of the first anterior dorsal fin are proportionally very smaller than the posterior ones such as in *Miguashaia* (Forey, 1998, fig. 11.13), *Coelacanthus granulatus* (Forey, 1998, fig. 11.4; Schaumberg, 1978, fig. 19), *Laugia* (Forey, 1998, fig. 11.10; Stensiö, 1932, pl. VIII), *Scleracanthus* (Stensiö, 1921, pl. 17 fig. 3) and *Rieppelia* (Ferrante & Cavin, 2023, fig. 2) or only slightly smaller such as in *Rhabdoderma elegans* (Forey, 1998, fig. 11.14) and *Foreyia* (Cavin et al., 2013, fig. S2).

#### Posterior dorsal fin

The basal plate of the posterior dorsal fin **(D2.b)** is unknown in PIMUZ T 3925 of *T. peyeri*, but it was scored as forked in Forey (1998) and subsequent works, probably because Rieppel (1980, fig. 6) figured this bone with a dotted line as forked in his reconstruction. The basal plate of the posterior dorsal fin is preserved in PIMUZ T 2651 and possibly also in PIMUZ T 978 (Fig. 13C, D). The basal plate of the posterior dorsal fin is composed of a fan-shaped distal plate and two elongated processes extending anteriorly and anteroventrally. The two processes form an angle of 40° between each other. The bone is then clearly forked.

Rieppel (1980) counted a number of 22 rays in the posterior dorsal fin of PIMUZ T 3925. In PIMUZ T 2651, we counted 13 rays plus 3 very small rays, but more rays might be present, because the fin is not completely prepared.

#### Anal fin

On PIMUZ T 978, a scattered forked basal plate is here interpreted either as the basal plate of the posterior dorsal fin or of the anal fin (Fig. 13E).

Rieppel (1980) counted 22 rays in the anal fin in PIMUZ T 3925 of *T. peyeri*. This fin is consequently the exact mirror of the posterior dorsal fin.

### Caudal fin and supplementary lobe

The caudal fin rays are described in the axial skeleton section above.

The supplementary lobe of the caudal fin is unknown in all specimens we examined (PIMUZ T 2651 and 3925). However, regarding the long fin rays in both lobes, and considering that the specimens are subadult or adult individuals, it is probable that the supplementary lobe would be included in the posterior profile of the caudal fin rather than developing beyond it.

### Ornamentation

#### Dermal bones

**Fig. 14:**
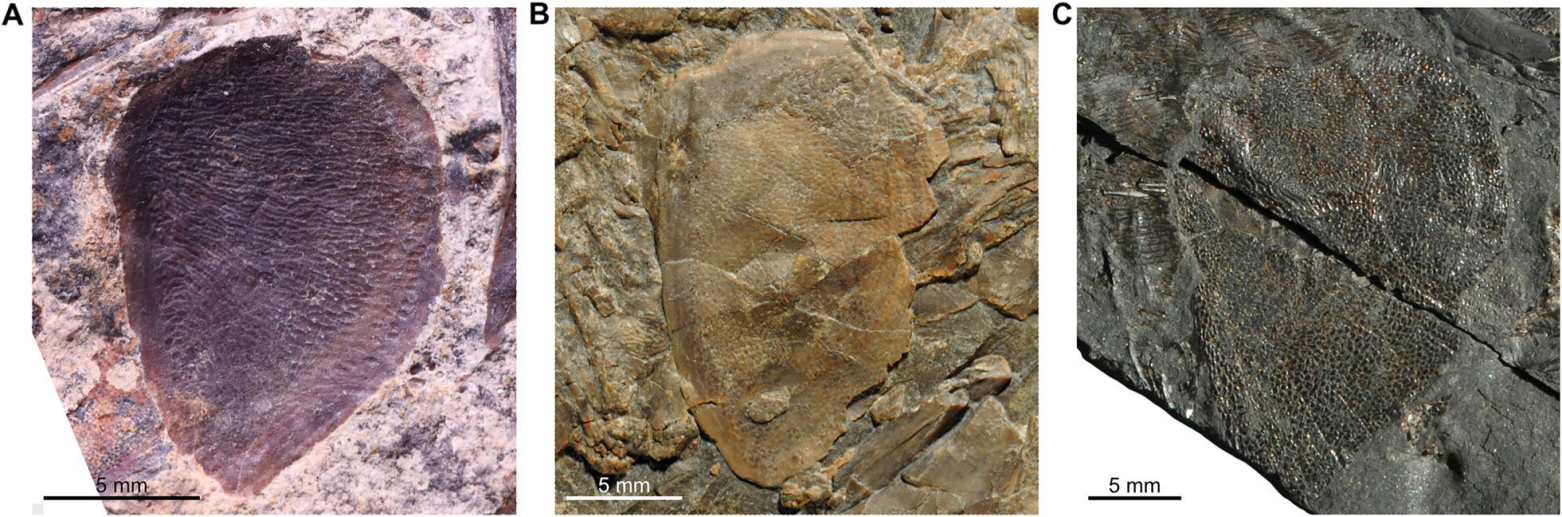
Opercles of *Ticinepomis peyeri* showing a progressive change in ornamentation with increasing size. Opercles of **A** PIMUZ T 978, **B** holotype PIMUZ T 3925a and **C** PIMUZ T 2653a (note that the image is mirrored for comparison). It can be observed that the ornamental pattern changes from a long wavy and linear tuberculation (**A**) to coarse round and closely spaced tubercles (**C**) with the increasing in size

In *T. peyeri*, the dermal bones of the skull roof, the cheek, and the angular present a strong tuberculate ornamentation, which shows variations in morphology among the different specimens. On PIMUZ T 3925, the postparietals, the preopercle, the opercles, and the angular are covered with coarse round tubercles (Figs 3A, 4, 5, 6A). The same kind of tubercular ornamentation is present in PIMUZ T 978 (angular and supraorbital; Fig. 6E), PIMUZ T 1513 (parietals and other unidentified dermal bones), and PIMUZ T 2653 (opercles and other unidentified dermal bones). In some other specimens, the dermal bones display tubercles with a different pattern. On PIMUZ T 978, which is a smaller individual compared to the two specimens mentioned above, the opercle is ornamented with long wavy ridges (Figs 9, 14A). This ornamentation covers the entire bone except a small posterior portion where the ridges develop as ovoid or round tubercles similar to those covering entirely the opercles of PIMUZ T 3925 (Fig. 14B) or PIMUZ T 2653 (Fig. 14C). The angular of PIMUZ T 978 bears the same long wavy ridges, but these, however, are discontinuous (Figs 3B, 9B-C) unlike on the angular of PIMUZ T 3925 that is covered with coarse round tubercles (Figs 3A, 4, 5). On PIMUZ T 2651, the supratemporal, the opercles, and other unidentified dermal bones are covered with discontinuous linear ridges that could also be qualified as elongated ovoid tubercles (Fig. 6C). Therefore, the dermal bones of the skull roof and the cheek of *T. peyeri* are ornamented by long wavy-to-linear continuous/discontinuous ridges evolving from coarse ovoid to round tubercles during ontogeny. A similar ornamentation with long wavy ridges is known mainly in Paleozoic coelacanths, such as *Serenichthys* (Gess & Coates, 2015) and in a few Mesozoic coelacanths, such as *Axelia* (Stensiö, 1921, pl. 16.6).

#### Scales

**Fig. 15:**
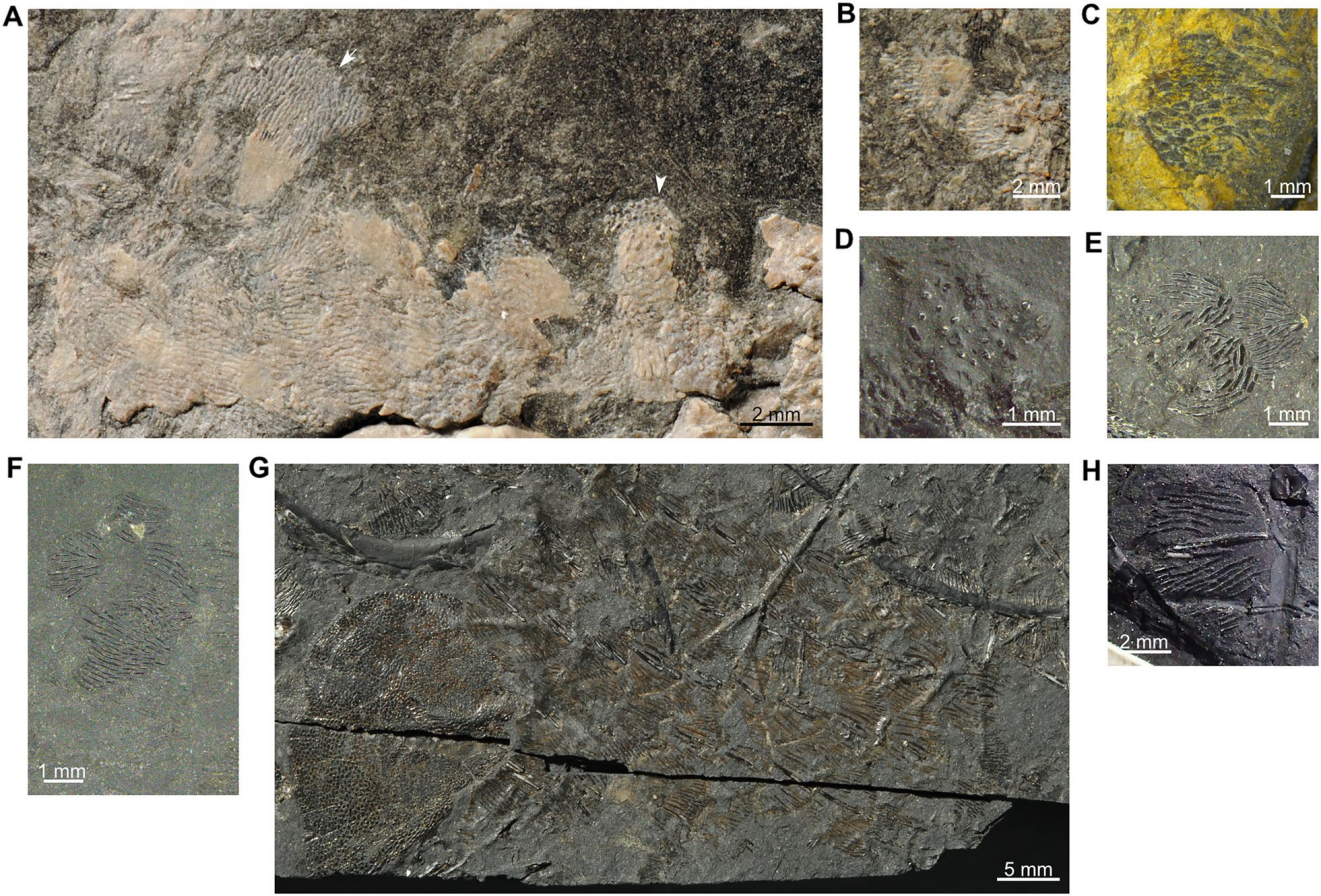
Scales of *Ticinepomis peyeri*. **A** Holotype (PIMUZ T 3925b), scales with longitudinal ridges (white arrow) located near the anterior dorsal fin, scales with round tubercles (white arrow-head) situated just behind the skull and **B** scales with ovoid tubercles located between the pectoral and pelvic fin. **C** PIMUZ T 1513, isolated scale. **D** PIMUZ T 2651a, scale (still partially covered by sediment) with round tubercles situated just behind the skull. **E**, **F** PIMUZ T 2651a, isolated scales. **G** PIMUZ T 2653a, scales of the flank presenting one or two elongated central ridges surrounded by many other smaller ridges. **H** PIMUZ T 2653a, isolated scale with two central elongated ridges surrounded by many smaller ridges

Rieppel (1980) described the scales of *T. peyeri* as having an exposed surface ornamented with closely packed and elongated ridges (“blunt or pointed spines”) disposed in a rostrocaudal direction. Regarding the different body areas, he found only little variation in the ornamentation of scales (Rieppel, 1980, fig. 7). However, it appears that the scales just behind the skull roof are covered with round tubercles unlike other scales in the body (Fig. 15A). Scales with round tubercles are also found in the same area behind the skull in PIMUZ T 2651 (Fig. 15D). In PIMUZ T 3925, the scales located between the pectoral and pelvic fins are covered with short and discontinuous ridges (Fig. 15B). Besides these variations, it seems that there is no other discernible variation in ornamentation of the scales between the different body areas. In PIMUZ T 2651, the few preserved scales show a pattern of ornamentation rather undifferentiated (Fig. 15E, F) and more similar to that of PIMUZ T 3925 (Fig. 15A). However, regarding PIMUZ T 2653, it appears that the ornamentation of scales through the body is not so uniformly distributed as in PIMUZ T 3925. Indeed, PIMUZ T 2653 shows a well-preserved flank with scales disposed in a natural position (Fig. 15H). The scales are here ornamented with small elongated and packed ridges flanking a longer and stouter central ridge (Fig. 15G). Scales similarly ornamented are known in some coelacanths, such as *Diplurus newarki* (Schaeffer, 1952, fig. 12) or *Heptanema paradoxum* (Renesto & Stockar, 2018, fig. 9) for instance. Therefore, it appears that in *T. peyeri* the ornamentation of scales cannot be characterised only as undifferentiated, according to Forey’s (1998) criteria, but should be regarded as polymorphic.

In PIMUZ T 3925, the openings for the lateral line in the scales cannot be observed because of their poor preservation. It should be stressed that, although correctly scored as unknown in Forey (1998), this feature of *Ticinepomis* has been erroneously scored with a known state since Dutel et al. (2012) and subsequent authors.

*T. ducanensis* sp. nov.

#### Diagnosis

*Ticinepomis* species of medium size characterised by the following unique combination of characters: premaxilla small; dentary simple developing as a splint-like rod; angular with numerous (at least fifteen) small and irregularly sized pores; splenial with eight pores including three anterior large pores plus five posterior small pores; splenial with a small symphyseal pore; angular unornamented and smooth with only faint ridges; basal plate of the anterior dorsal fin with a straight ventral margin, an anterodorsal margin dug by a notch and no posteroventral spine; basal plate of the posterior dorsal fin with two anterior processes forming an angle of 50°; pelvic bones with robust processes.

#### Measurements and meristic

Total body length: 615 mm (estimation); d1.f = 10; pect.f ≥ 18; n.a = 29–49 (maximal estimation); h.a≥ 12.

#### Etymology

The species name refers to the mountain Ducan Dador/Gletscher Ducan, which is nearby the locality where the holotype was found.

#### Holotype

PIMUZ A/I 2985, a sub-complete specimen of 615 mm length (estimation) with the entire skull preserved in ventral view and with the axial skeleton, including basal plates of the anterior and posterior dorsal fins, the pectoral girdle and the pelvic basal plate; Ducanfurgga 10 (Canton Graubünden, Switzerland); middle part of the Prosanto Formation, latest Anisian or earliest Ladinian (Middle Triassic).

#### Referred material

PIMUZ T 435, specimen showing bones of the lower jaw, cleithrum, gular plates, some teeth of the basibranchial apparatus and some neural arches; Point 902/Mirigioli, Meride (Canton Ticino, Switzerland); bed 158, upper Besano Formation, *E. curionii* Ammonoid Zone, earliest Ladinian (Middle Triassic).

#### Localities and horizons

Ducanfurgga 10 (Canton Graubünden, Switzerland) and Monte San Giorgio (Canton Ticino, Switzerland), middle part of the Prosanto Formation, latest Anisian or earliest Ladinian (Middle Triassic) and upper Besano Formation, Early Ladinian (Middle Triassic).

#### Nomenclatural act

The present work and its nomenclatural act are registered in ZooBank, the online registration system for the International Commission on Zoological Nomenclature. The Life Science Identifiers for this publication is “urn:lsid:zoobank.org:act:ECEACC38-A343-44C2-9201-DCD1C7AFA01E” and can be resolved appending the prefix “http://zoobank.org/” in any standard web browser.

## Description of *Ticinepomis ducanensis* sp. nov.

### Generalities

**Fig. 16:**
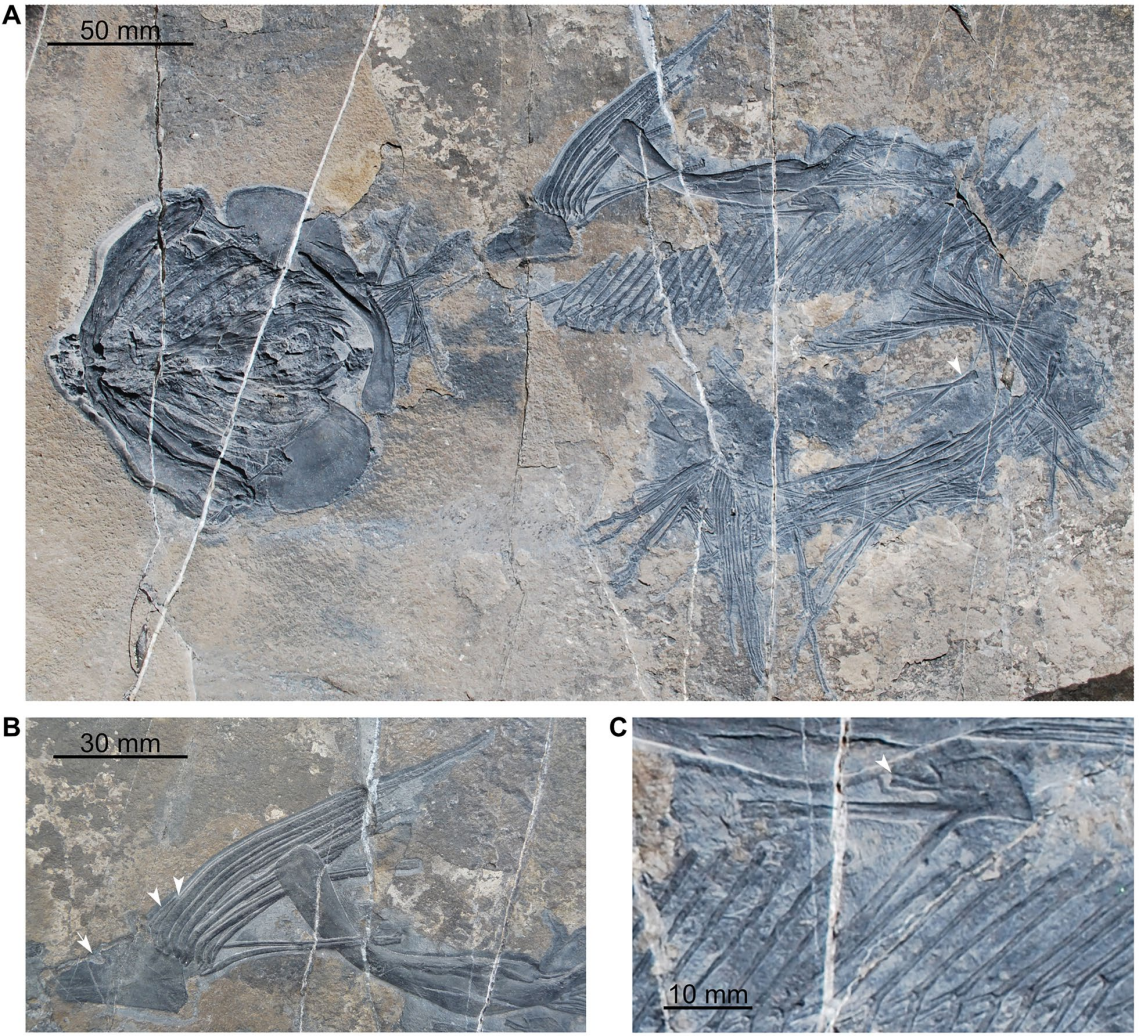
*Ticinepomis ducanensis* sp. nov. (holotype A/I 2985). **A** Entire skeleton. The basal plate of the anal fin is indicated by a white arrowhead. **B** Close-up view of the anterior dorsal fin in left lateral view showing the anterodorsal notch (white arrow) on the basal plate and the two anterior smaller fin rays (white arrowhead). **C** Enlargement of the basal plate of the posterior dorsal fin in left lateral view showing the small anteriorly directed process (white arrowhead)

**Fig. 17:**
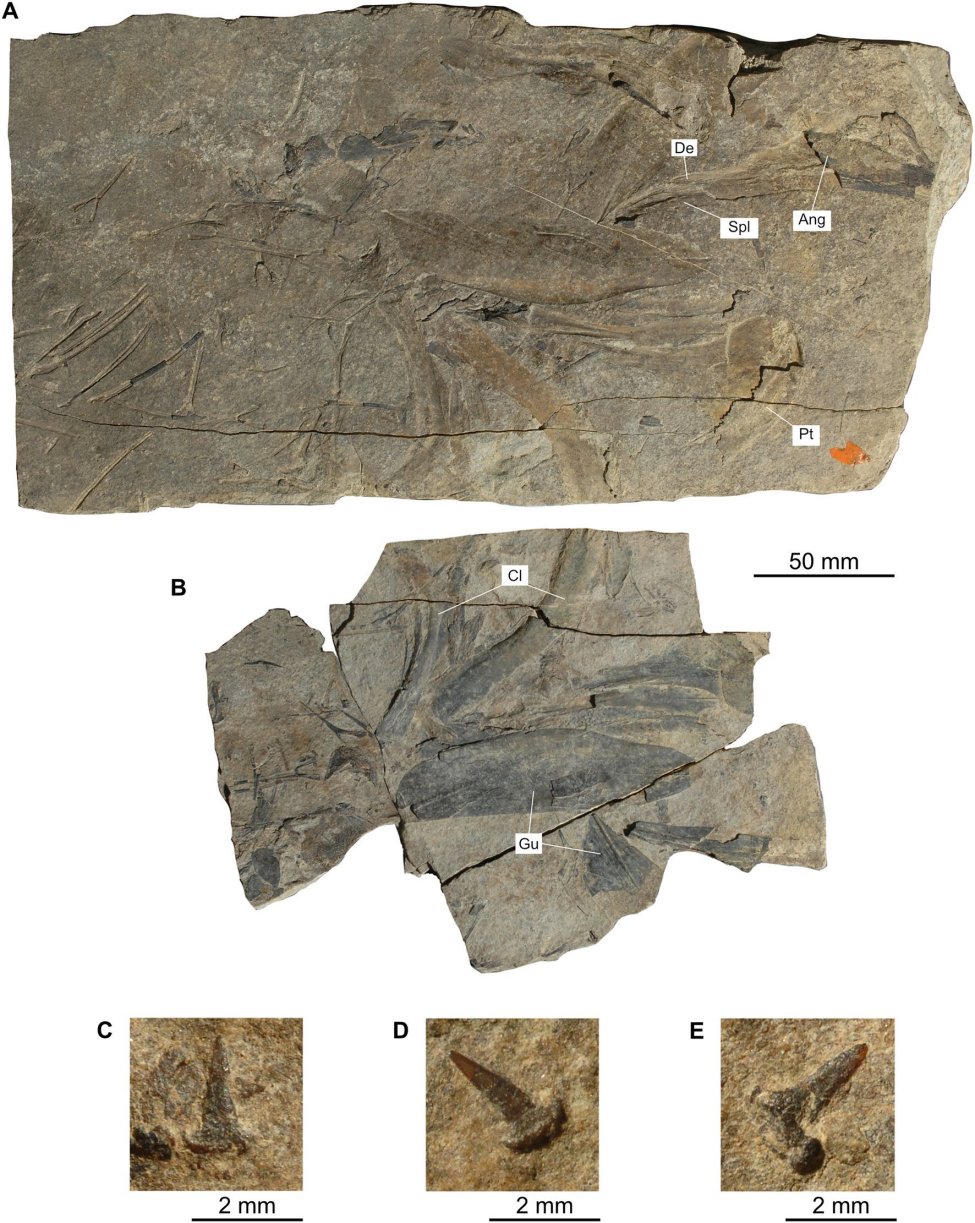
*Ticinepomis ducanensis* sp. nov. (PIMUZ T 435)**.** Photograph of **A** the part and **B** counterpart. **C-E**, Isolated branchial teeth

A detailed description with photographs of the holotype (PIMUZ A/I 2985) is provided in Cavin et al. (2013, figs 4, 5, 6). Here, we describe the specific characters that allow distinguishing the new species from the type species *Ticinepomis peyeri*. The new species *T. ducanensis* sp. nov. is currently known from a sub-complete specimen and a partial specimen, namely the holotype PIMUZ A/I 2985 (Figs 3E,F, 16) and the specimen PIMUZ T 435 (Figs 3D, 17), respectively. The anatomical features of the latter specimen, especially those of the lower jaw, are similar to those observed on bones of PIMUZ A/I 2985, allowing to refer PIMUZ T 435 to the new species *T. ducanensis*.

*Ticinepomis ducanensis* sp. nov. is characterised by its larger size, reaching an estimated length of circa 615 mm (Fig. 16; Cavin et al., 2013), which distinguishes it from the type species *T. peyeri* reaching an adult size of about 180 mm long. The holotype (PIMUZ A/I 2985) may represent an adult individual as all the basal plates are fully ossified, which is a feature observed in adult coelacanths (e.g., Schultze, 1980; Witzmann et al., 2010). The presence in this specimen of a fully ossified axial mesomere and scapulocoracoid (Cavin et al., 2013, fig. 5) reinforces this conclusion.

### Dermal bones of the skull

The skull roof of *T. ducanensis* sp. nov. is mostly unknown as it is preserved in ventral aspect (Cavin et al., 2013). The tip of the snout appears to be composed of small elements, including a pair of premaxillae and rostral elements. The premaxilla bears two large teeth plus two smaller ones as in *T. peyeri*, but the bone is smaller than the large premaxilla of *T. peyeri*. However, it should be emphasized that this observation may be biased by their preservation in ventral aspect. Posterior to the premaxillae is a single, small median rostral ossicle (Cavin et al., 2013). Globally, the snout of *T. ducanensis* sp. nov. appears to be less robust than the one of *T. peyeri*.

### Dermal bones of the cheek

From the cheek bones and opercular portion, only the preopercles and opercles are known. The identified preopercles are triangular shaped (Cavin et al., 2013, fig. 5), recalling somewhat the identified preopercle of *T. peyeri*. The opercles are ovoid-shaped, with an almost straight dorsal border and slightly curved anterior and posterior borders (Cavin et al., 2013, fig. 5), being then clearly reminiscent of that of *T. peyeri*.

### Lower jaw

The lower jaw is the most distinctive anatomical element of *T. ducanensis* sp. nov. presenting some similarities and differences with *T. peyeri*. The organisation of the lower jaw is comparable to that of other coelacanths and includes a retroarticular and an articular represented as separated bones, a prearticular, an angular, a dentary, and a splenial, which are all identified in PIMUZ A/I 2985 and in PIMUZ T 435. The lower jaw is shallow and parallel-sided producing then a long and shallow lower jaw, which is peculiar among coelacanths but is reminiscent of *T. peyeri*, for instance.

The angular **(Ang)** is the largest element of the jaw. The surface of the angular is smooth with no tubercular ornamentation and bears only faint ridges (Figs 3E, F, 16A; Cavin et al., 2013, fig. 5). The mandibular sensory canal **(m.s.c)** opens within the angular through a series of many irregularly sized pores occurring as shallow grooves oriented backwards in the posterior region and frontwards in the anterior region. In the holotype, 6–7 pores at least are located in the middle of the angular, but there are more pores posteriorly. On specimen PIMUZ T 435, a series of at least ten pores extends from the posterior portion to the middle of the bone. Based on these observations, 15 small pores at least open on the angular. On the boundary of the retroarticular and angular (PIMUZ A/I 2985) is a large and elongated pore, which corresponds to the entrance of the subopercular branch of the preopercular sensory canal **(sop.br)** (Fig. 3E,F; Cavin et al., 2013, fig. 5b). The latter feature is currently known only in latimeriid coelacanths, namely in *Megalocoelacanthus* (Dutel et al., 2012), *Libys*, *Macropoma*, *Holophagus*, and *Latimeria* (Forey, 1998).

The splenial **(Spl)** is strongly anteriorly recurved downwards, which is reminiscent of *T. peyeri* (Figs 3C, F, 16A). On the symphyseal margin of the splenial is a notch forming, when in contact with its antimere, a symphyseal pore. In *T. ducanensis* sp. nov., the pore is, however, smaller than in *T. peyeri* (Fig. 3C, F). On the splenial, eight pores are present for the mandibular sensory canal, including three anterior large pores plus five posterior small pores (Fig. 3F; Cavin et al., 2013). The shape and the total number of pores in the lower jaw of *T. ducanensis* sp. nov. is then different from *T. peyeri* (Fig. 3C, F).

The dentary **(De)** of *T. ducanensis* sp. nov. is characteristic. The bone develops as an elongated and narrow splint-like bar (Figs 3E, F, 16A), which is different from the strongly hook-shaped dentary of *T. peyeri*, and thus represents a specific variation. Furthermore, it should be pointed out that a simple dentary is currently unique among the Latimerioidei. The dentary of *T. ducanensis* sp. nov. forms a pronounced ventral angle midway along its length, similar to that of *T. peyeri*. The dentary bears a small dentary pore.

Three coronoids have been reported and illustrated in *T. ducanensis* sp. nov. by Cavin et al. (2013, fig. 5). However, this assumption is here questioned, because their emplacement on the oral margin of the lower jaw is odd. Indeed, the first two coronoids are found directly placed upon the splenial and the most posterior one just anterior to the middle point above the dentary (Cavin et al., 2013, fig. 5b). Considering this arrangement, it is likely that the first anterior coronoid, which forms the dorsal portion of the symphyseal margin, is rather a small ossified mentomeckelian, also known in some coelacanths such as *Whiteia*, *Laugia*, or *Latimeria* (Forey, 1998) for instance. The second coronoid, posterior to the latter bone, is probably a coronoid but one that had been shifted from its natural position. It is therefore unlikely that there were only three coronoids in *T. ducanensis* sp. nov., a feature currently known only in *Diplocercides* (Forey, 1998). The exact number of coronoids in the genus *Ticinepomis* remains unknown.

The gular plates are unknown in PIMUZ A/I 2985 and are known only in PIMUZ T 435. The gular plates **(Gu)** are large bones (Fig. 17A, B). The anterior margin along the midline is pointed, while the posterior margin is more rounded. The lateral margin is convex, slightly swollen on its anterolateral margin, and mesial margin is almost straight, giving to the bone a parallel-sided shape. A pronounced ridge runs parallel to the lateral edge along the anteroposterior axis. According to Dutel et al. (2012), this ridge, observed in *Megalocoelacanthus* and *Latimeria* for instance, corresponds to the insertion point of the anterior and posterior rami of the intermandibular muscle. The surface of the gular plates is smooth and unornamented. The gular plate of *T. ducanensis* sp. nov. (Fig. 17A,B) is clearly reminiscent of the one of *T. peyeri* (Figs 5, 9B-C)*.*

### Neurocranium and gill arches

The neurocranium is composed of a pair of prootic, a basioccipital and an exoccipital (opisthotic), all ossified as independent bones (Cavin et al., 2013). The prootic extend back forming a posterior wing that sutures with the basioccipital in a more or less wavy complex suture. Although difficult to assess, there is indication that the otico-occipital portion is significantly shorter, probably less than half than the ethmosphenoid portion (Cavin et al., 2013).

Fragments of basibranchial tooth plates have been recognised, including a large median plate with at least three smaller plates located laterally (Fig. 16A; Cavin et al., 2013, fig. 5), a configuration reminiscent to that of *Whietia woodwardi* (Forey, 1998, fig. 7.6D).

The branchial apparatus is well known in PIMUZ A/I 2985 (Cavin et al., 2013). It is composed of five pairs of ceratobranchials and one pair of ceratohyals. The ceratobranchials of *T. ducanensis* are covered with tooth plates, not well visible, and larger spaced conical teeth, 1.5–2 mm high, borne above a conspicuous basal support (Cavin et al., 2013). Those teeth are observed in PIMUZ A/I 2985 and PIMUZ T 435 (Fig. 17C–E; Cavin et al., 2013, figs 5, 6). The exact outline of the ceratohyal is not known precluding comparison with *T. peyeri*. However, the ventral process, which is well developed, is located at the mid-length of the bone, similar to that of *T. peyeri*.

An urohyal, fragments of basibranchial tooth plates, pairs of symplectics and of interhyals are also known in PIMUZ A/I 2985 (Cavin et al., 2013).

### Axial skeleton

The axial skeleton of *T. ducanensis* (PIMUZ A/I 2985) is incomplete and the exact number of neural arches is unknown (Cavin et al., 2013). A number of 28 neural arches are preserved in anatomical position from the level of the basal plate of the anterior dorsal fin to beyond the posterior basal plate of the posterior dorsal fin with one additional arch preserved very close to the skull (Fig. 16A; Cavin et al., 2013). Based on the disposition of the specimen, less than ten neural arches are missing anteriorly to the basal plate of the first dorsal fin. The preserved posterior-most arches represent the first arches of the caudal region as indicated by their enlarged distal portion, their position compared to the basal plate of the posterior dorsal fin and the presence of haemal arches ventrally (Cavin et al., 2013). It is likely that no more than ten neural arches are missing in the caudal portion. Therefore, considering the missing anterior and posterior neural arches, it can be hypothesised that the actual number of neural arches should have been lower than 50. There are at least 12 haemal arches and no ossified elongated ribs in the abdominal area of *T. ducanensis* (Fig. 16A; Cavin et al., 2013).

### Paired fins

#### Pectoral girdle and fins

The pectoral girdle includes a cleithrum, a scapulocoracoid, an extracleithrum, and a clavicle (Cavin et al., 2013). The cleithrum of *T. ducanensis* sp. nov., known in PIMUZ A/I 2985 (Fig. 16A) and in PIMUZ T 435 (Fig. 17A, B), develops as a large and robust bone with an expanded dorsal extremity, reminiscent of *T. peyeri*. The clavicle presents traces of a ridged ornamentation. The anocleithrum remains unknown.

##### Pelvic girdle and fins

The pelvic bones present a posterior, a lateral and a medial process, the latter extending posteriorly as a wing-like structure (Fig. 16A; Cavin et al., 2013, fig. 4). However, the global shapes (i.e., the processes) of the pelvic bones of *T. ducanensis* sp. nov. appear more robust than the pelvic bones observed in *T. peyeri* (Fig. 13F).

### Unpaired fins

#### Anterior dorsal fin

The basal plate of the anterior dorsal fin is large and approximately triangular in shape. Its ventral margin is straight and its posterior margin is slightly curved. The anterodorsal margin is dug by a notch (Fig. 16B; Cavin et al., 2013), which is absent in *T. peyeri*. In the Anisian Dont Formation (Northern Dolomites, Italy), an indeterminate and poorly known coelacanth (PZO 625) thought to be a relative of *Foreyia* and *Ticinepomis* shows a somewhat similar notch located on the anterodorsal margin (Renesto & Kustatscher, 2019, fig. 4A-B).

The anterior dorsal fin of *T. ducanensis* sp. nov. has eight long segmented rays preceded by two smaller unsegmented rays that are ornamented with denticles (Fig. 16B; Cavin et al., 2013, fig. 4). This configuration (i.e., number and shape of rays) of the anterior dorsal fin is reminiscent of *T. peyeri* that has eight long segmented rays preceded by 1–2 smaller unsegmented ray(s), and of the coelacanth from the Dolomites (PZO 625) that has ten long segmented rays preceded by one smaller ray (Renesto & Kustatscher, 2019). The latter coelacanth is, however, different from *T. ducanensis* sp. nov., because the fin rays bear larger and more developed denticles. However, this difference does not appear to be ontogeny related as the type specimen of *T. ducanensis* sp. nov. is considerably larger than the coelacanth from the Dolomites that appears to have a length similar to that of *T. peyeri*.

#### Posterior dorsal fin

The basal plate of the posterior dorsal fin is formed by a main plate and two elongated processes extending anteriorly and anteroventrally at an angle of 50° between each other (Fig. 16C; Cavin et al., 2013). This angle is close to, but different from the coelacanth from the Dolomites, which has both elongated processes forming an angle of 55° (Renesto & Kustatscher, 2019). The angle is also considerably different from the one in *T. peyeri* that presents an angle of 40° between the two processes. In *T. ducanensis* sp. nov.*,* the anterodorsal corner of the main plate extends anteriorly above the upper anterior process as a small process (Fig. 16C; not figured in Cavin et al., 2013, fig. 4). Such a process, although different in shape and size, is observed also in *Latimeria* (Millot & Anthony, 1958, pl. LIXa), *Laugia* (Forey, 1998, figs 8.3a and 8.3c), *Guizhoucoelacanthus* (Geng et al., 2009, fig. 1) and potentially in *Piveteauia* (Clément, 1999).

#### Anal fin

The basal plate of the anal fin, preserved in dorsal or ventral view, (Fig. 16A; drawn but not labelled and described in Cavin et al., 2013, fig. 4) is formed by a main plate with two elongated processes extending anteriorly.

## Discussion

### Paleoecology and paleobiology of *Ticinepomis*

#### Diet

Several authors (e.g., Forey, 1998; Meunier et al., 2018; Uyeno & Tsutsumi, 1991; Zatoń et al., 2017) have investigated the diet of living and extinct coelacanths. The living coelacanth *Latimeria* is considered a solitary and nocturnal predator essentially feeding on benthic fishes and occasionally on cephalopods (e.g., Uyeno & Tsutsumi, 1991). When a prey is detected probably by its rostral organ system (e.g., Forey, 1998), *Latimeria* rapidly opens its mouth to grasp the prey and swallow it very quickly, without chewing it (Meunier et al., 2015). This is supported by the stomach contents of captured *Latimeria* that reveal almost undamaged prey (Uyeno & Tsutsumi, 1991). Moreover, the teeth and especially the fangs of *Latimeria* are rather adapted to retain the prey and prevent them from escaping from the mouth cavity (Meunier et al., 2015).

Most extinct species of coelacanths had a dentition comparable to that of *Latimeria*. On the pterygoids and prearticulars, teeth are generally small rounded or conical, while, on the parasphenoid, the teeth are slightly larger and bulbous. By comparison, on the dermopalatines, ectopterygoids, coronoids, and dentaries, the teeth are thinner, larger, and more pointed, some of them being described as fang-like in some taxa (*Latimeria*). Fossilised remains from the digestive tracts of coelacanths indicate a variety of prey, such as conodont elements within a specimen of cf. *Diplocercides* (Zatoń et al., 2017), an intact paleostomatopod shrimp in a specimen of *Caridoscutor* (Lund & Lund, 1985), a small incomplete, crushed crustacean in a specimen of *Swenzia* (Clément, 2005) and actinopterygian fish remains in a specimen of *Axelrodichthys araripensis* (Meunier et al., 2018). A specimen of a *Coccoderma* sp. (BSP 2002 I 36) in the collection of the Bayerische Staatssammlung für Paläontologie und Geologie (Munich, Germany) contains an entire shrimp stuck in the mouth cavity. The position of the shrimps within the mouth shows that the fish likely died when trying to swallow its prey. According to Forey (1998), some coelacanths present more rounded and striated teeth, corresponding to a crushing dentition, such as *Spermatodus*, *Axelia*, *Libys*, *Axelrodichthys*, and *Mawsonia*, to which we can add *Megalocoelacanthus* (Dutel et al., 2012). Forey (1998) regarded this condition as derived compared to coelacanths with small, villiform, and smooth teeth adapted for both grasping and holding preys.

*Ticinepomis peyeri* has different kinds of teeth in the buccal cavity. The conical and not striated teeth borne on the coronoid and the dermopalatine are the largest (Fig. 10A, B). The prearticular, pterygoid, and ectopterygoid (Figs 10C, 12) are densely covered with round, small conical teeth that are finely striated, with the exception of those on the ectopterygoid. The surface of the parasphenoid is toothed on its anterior two-thirds with large-to-small, strongly conical and striated teeth (Figs 10D, 11). The dentition of *T. peyeri*, formed by strong, mostly bulbous teeth, is likely related to a specialized diet, namely a durophagous feeding behavior. We hypothesise that its stout dentition served to crush prey with hard body parts, such as carapaces of crustaceans or shells of molluscs. Moreover, the relatively small size of *T. peyeri* indicates that it was preying on small animals, such as small crustaceans, bivalves, and snails inhabiting the shallow margins of the basin (Furrer & Vandelli, 2014). Nevertheless, the competition may be high, because the basin was inhabited by other animals having also strong crushing dentition, such as the actinopterygians *Archaeosemionotus*, *Colobodus*, and *Crenilepis* and, marine reptiles, as the placodont *Cyamodus hildegardis* and the ichthyosaur *Mixosaurus kuhnschnyderi* (Furrer & Vandelli, 2014).

#### Ontogeny and sexual dimorphism

As described above, the ornamentation of dermal bones shows strong pattern variations depending on the size of the bones, and therefore on the body size of the individuals to which they belong. Such progressive changes in ornamentation with increasing size have already been recorded in specimens of *Serenichthys*, a coelacanth from the Famennian (Upper Devonian) of South Africa (Gess & Coates, 2015). In this genus, the ornamentation of dermal bones, in particular of the opercles, evolves from long wavy ridges in young individual into elongate tubercles in older individuals (Gess & Coates, 2015). The ornamentation of *T. peyeri* is subject to a comparable change of its ornamentation according to the age of the individual, and may provide an indication on the ontogenetic stage of specimens. In PIMUZ T 978, the gular pit line is long and well developed. It has been proposed that the length of the gular pit line is linked to the ontogenetic stage and that the length of the pit line, which stays of the same length through life, tends to become proportionally smaller compared to gular plate size during ontogeny (Hensel, 1986). Together with the ornamentation of long wavy ridges covering this bone, the long gular pit line reinforced the hypothesis that PIMUZ T 978 is a young individual. The changes in of ornamentation during ontogeny should cause caution when using characters based on the typology of the ornamentation in phylogenetic analyses. However, we consider that the use of ornamentation in phylogenetic analyses still is a reliable character to distinguish species of coelacanths if the character states are carefully defined based on adult individuals.

Because younger individuals of *T. peyeri* are found together with adult individuals, this implies that individuals of different ages probably inhabited the same areas of the basin, with no apparent segregation between juveniles and adults. The situation is reversed with respect to the Devonian *Serenichthys* assemblage. Indeed, *Serenichthys* was recovered from an estuarine environment, which is interpreted as a safe spawning and nursery ground for a diverse fish fauna due to the scarcity of adult elasmobranchs and the lack of non-juvenile coelacanths (Gess & Coates, 2015).

Comparing the different specimens of *T. peyeri*, it appears that the scales show a variation in ornamentation between individuals. PIMUZ T 2651, 2653, and 3925 represent individual of the same ontogenetic stage (i.e., almost adult individual) as indicated by their ossification rate, body length, and kind ornamentation of the dermal bones. In PIMUZ T 2651 and 3925, scales present a pattern of ornamental undifferentiated with no clear central ridge (Fig. 15A, E, F). Conversely, PIMUZ T 2653 has scales clearly differentiated with a central ridge (Fig. 15G, H). Thus, the apparent variation of ornamentation of the scales between these three specimens is better explained by sexual dimorphism rather than by ontogeny.

### Systematic relationships of *Ticinepomis* species with other coelacanths from the Triassic of Europe

The systematic position of the Swiss Triassic coelacanth taxa *Ticinepomis*, *Foreyia,* and *Heptanema* has been assessed in recent phylogenetic analyses (e.g., Cavin et al., 2013, 2017; Renesto & Stockar, 2018; Renesto et al., 2021; Toriño et al., 2021). In these works, *Ticinepomis* is resolved as a member of the Latimeriidae, while the position of *Heptanema* remains unsure. Following the description of *Rieppelia* (Ferrante & Cavin, 2023), *Ticinepomis* was resolved as the sister to the pair *Foreyia* - *Rieppelia.*

Beside the Swiss coelacanths from the Monte San Giorgio (Ferrante et al., 2017; Renesto & Stockar, 2018; Renesto et al., 2021; Rieppel, 1980, 1985) and from the Ducan-Landwasser region near Davos (Cavin et al., 2013, 2017), other coelacanths have been described from the Triassic of Europe, such as *Dobrogeria aegyssensis* (Cavin & Grădinaru, 2014) from the Olenekian (Early Triassic) of North Dobrogea (Romania), *Garnbergia ommata* (Martin & Wenz, 1984) from the Upper Muschelkalk (Ladinian, Middle Triassic) of Baden-Württemberg (Germany), *Hainbergia granulata* (Schweizer, 1966) from the Upper Muschelkalk (Ladinian, Middle Triassic) of Göttingen (Germany), *Alcoveria brevis* (Beltan, 1972 and 1984) from the Muschelkalk (Ladinian, Middle Triassic) of Alcover (Spain), *Heptanema paradoxum* (Belloti, 1857) from the late Ladinian (Middle Triassic) Perledo-Varenna Formation of Lombardy (Italy), *Graphiurichthys callopterus* (Kner, 1866) from the Carnian (Late Triassic) of Raibl (Italy), *Coelacanthus lunzensis* (Teller, 1891) from the Carnian (Late Triassic) of Lunz-am-See (Austria), and *Urocomus picenus* (= ”*Undina picena”*) (Bassani, 1896; Costa, 1862) from the Norian (Late Triassic) Dolomia Principale di Giffoni (Province of Salerno, Italy).

Apart from these coelacanths identified at a specific level, other coelacanth material is currently not identified at generic or specific levels, we could mention a relative of *Foreyia* from the Anisian (Middle Triassic) Dont Formation of Northern Dolomites in Italy (Renesto & Kustatscher, 2019), a complete but poorly preserved fossil from the Anisian (Middle Triassic) of the Massif des Vosges in France (Gall et al., 1974), several disarticulated bones representing different individuals from the Ladinian (Middle Triassic) of the Schwäbisch Hall Formation of Baden-Württemberg, Germany (Hagdorn & Mutter, 2011) and remains of mawsoniid coelacanths from Rhaetian deposits (Late Triassic) of France (Deesri et al., 2018) and Germany (Hartung et al., 2021). From this list of Triassic coelacanths, we only compare those that share similarities with *Ticinepomis*.

Historically, the very first fossil of coelacanth found at Monte San Giorgio was identified and described in 1916 by the Swedish paleontologist Erik H. O. Andersson, later known as Stensiö. This material, not examined here, is represented by a slab with scattered bones and remains of scales of a small coelacanth from ‘Cava Tre Fontane’ (Andersson, 1916). The description and the illustration by Andersson (1916, pl. 3.1–2) provide little information, but the description of the ornamentation of the scales and the shape of the cleithrum seems to point to the genus *Ticinepomis* Rieppel, 1980. According to the ornamentation of the scales, Andersson (1916) referred this material to “*Undina* sp.”, a genus that was then known by two specimens from the Late Triassic of Giffoni (Province of Salerno, Italy). The first fossil from Giffoni, a fragmentary caudal fin of coelacanth, was named *Urocomus picenus* by Costa (1862). Bassani (1896) then described under the name “*Undina picenus*” (“*Undina picena*” as amended by Forey, 1998) a second specimen represented by a nearly complete but very badly preserved coelacanth specimen recovered from the Norian (Late Triassic) from Giffoni (Valle Piana locality, Province of Salerno, Italy). Rieppel (1980) considered the specimens of “*U. picena*” described by Costa (1862) and Bassani (1896) to be closely similar to *Ticinepomis peyeri*. According to the illustration provided by Bassani (1896, pl. 11.1), the fossil is roughly reminiscent of *Ticinepomis*. “*U. picena*” shows a rounded opercle as in *Ticinepomis* and, according the short description provided by Forey (1998), all scales are ornamented with regular-sized rounded tubercles. However, as already mentioned by Rieppel (1980), “*U. picena*” appears to be different to some degree. The posterior dorsal fin of “*U. picena*” is composed of 12 rays, while in *T. peyeri*, there are 22 rays. Rieppel (1980) claimed that the larger size of “*U. picena*”, being 300–350 mm long, compared to *T. peyeri* is a distinctive difference between both species. We agree with Rieppel (1980) that the size of the holotype of *T. peyeri*, reaching 180 mm, is a specific feature, which is clearly different from “*U. picena*”. Rieppel (1980) found that the scales of “*U. picena*” present shorter spines (Bassani, 1896, pl. 15.63) compared to *T. peyeri*. The latter difference needs to be taken with caution, because it could also be related to different ontogenetic stages of the specimens. The basal plate of the second dorsal fin has its two elongated processes forming an angle of 50° (Bassani, 1896, pl. 15.60), which is different from *T. peyeri* that has the two elongated processes forming an angle of 40°. Although the angle in “*U. picena*” is similar to that of *T. ducanensis* sp. nov., the main plate develops in a considerably different way. Moreover, the pelvic bones of “*U. picena*” (Bassani, 1896, pl. 15.56) are also differently shaped than the ones of *T. peyeri* and *T. ducanensis* sp. nov. The available descriptions and illustrations provided by Costa (1862) and Bassani (1896) are of little help to assess the position of “*U. picena*”. Rieppel (1980) also considered the descriptions and illustrations provided by these authors to be insufficient to allow proposing a generic synonymy with *T. peyeri*, although he admitted that a re-examination of the Italian fossils could possibly lead to a generic synonymy. As a conclusion, the specimens described by Costa (1862) and Bassani (1896) under the name “*U. picena*” are probably related to the genus *Ticinepomis* but represents likely a distinctive species. If this relationship is confirmed, it would potentially increase the stratigraphic range of *Ticinepomis*, from the Anisian to the Norian, i.e., about 40 million years, but without increasing considerably its geographic distribution.

Hagdorn and Mutter (2011) reported from the Ladinian Serrolepisbank of the Schwäbisch Hall Formation (Baden-Württemberg, Germany) a medium-sized to large actinistian reaching an estimated length of 1.3 m. The material is composed of several isolated bones that represent potentially different growth stages of a single species according to the authors. Some bones have been figured with high-quality photographs by Hagdorn and Mutter (2011, fig. 7), the rest of the material awaiting description. Some of the figured bones show interesting similarities with *T. ducanensis* sp. nov. The angular (Hagdorn & Mutter, 2011, fig. 7h) is of the same shape and has a smooth surface with no tubercular ornamentation and only faint ridges. The shape of the opercle (Hagdorn & Mutter, 2011, fig. 7e) is also round-to-roughly triangular. The urohyal (Hagdorn & Mutter, 2011, fig. 7f) presents a very similar shape as the one of *T. ducanensis* sp. nov. The dentition of the parasphenoid (Hagdorn & Mutter, 2011, fig. 7i) is reminiscent to that observed in *T. peyeri* (Figs 10, 17D) and consists of large conical teeth that decrease in size posteriorly along the external margin, and smaller teeth along the median portion of the bone. The scales (Hagdorn & Mutter, 2011, fig. 7i) are ornamented with a pack of elongated ridges as in *T. peyeri*. Considering the previous short comparison, the coelacanth taxon from the Serrolepisbank is different from *T. peyeri*, but it is possible that it represents either a genus related to *Ticinepomis* or a distinct species of *Ticinepomis*. According to Hagdorn and Mutter (2011), the facies and the fauna of the Serrolepisbank gives evidence of a lacustrine environment. Therefore, this coelacanth taxon is of great interest, because Triassic freshwater coelacanths are rare and currently only known from the United States with *Quayia* (Hunt, 1997; Johnson et al., 2002), *Moenkopia* (Schaeffer & Gregory, 1961), and the mawsoniids *Chinlea* (Elliott, 1987; Schaeffer, 1967) and *Diplurus* (Schaeffer, 1952).

Teller (1891) described *Coelacanthus lunzensis*, a sub-complete specimen with a poorly preserved head, from the Carnian (Upper Triassic) freshwater deposits of Lunz-am-See (Austria). According to the description and illustrations of Reis (1900, pls 9, 10), *C. lunzensis* presents some interesting similitudes with *Ticinepomis* spp. but also some striking differences. The basal plate of the anterior dorsal fin of *C. lunzensis* is roughly triangular shaped recalling the one of *T. ducanensis*, except that, in first, there is no notch on the anterodorsal margin. The anterior dorsal fin is composed of ten rays (Reis, 1900). Regarding the illustration of *C. lunzensis* (Reis, 1900, pl. 9), it appears that the first anterior ray is smaller than the posteriorly following rays. The latter aspect and number of rays is reminiscent of *Ticinepomis* species. The axial skeleton of *C. lunzensis* is composed of at least 42 neural arches, including 18 arches in the caudal region (Reis, 1900). This proportionally short body and low number of neural arches (less than 50) is reminiscent of *Ticinepomis*. The bones of the skull roof are densely ornamented with round small tubercles (Reis, 1900), which is an ornamentation pattern reminiscent of that of *T. peyeri*. However, *C. lunzensis* is different from *Ticinepomis* spp. by some striking anatomical features. *C. lunzensis* possesses a large ossified lung (ossified swim bladder of Reis, 1900), which is clearly absent in *Ticinepomis* spp. The principal coronoid (preoral coronoid of Reis, 1900) is large and, according to Reis (1900), is reminiscent of *Coccoderma gigas* Reis 1888, while this bone is shallow in *Ticinepomis* spp. It should be warned that Reis (1900) considered the attribution of *C. lunzensis* to the genus *Coelacanthus* as ‘temporary’ and that this species should eventually be included in another genus, because, according to him, this specimen is more closely related to Jurassic coelacanth genera than to genera of older periods. We agree with Reis (1900) that *C. lunzensis* appears to be more closely related to other derived coelacanths, especially to Triassic coelacanths, and is not related to the Permian genus *Coelacanthus*.

This overview of coelacanth taxa from the Triassic of Europe demonstrates that their diversity and their morphological disparity were high during this period. Moreover, coelacanths also occupied a wide variety of ecological niches in both marine and freshwater environments. Considering the *Ticinepomis* species from Switzerland, it appears that some other coelacanth taxa are potentially related in some degree to this genus. After the Permian–Triassic mass extinction, coelacanths experienced a high peak in taxic diversity in the Early Triassic and to a lesser degree in the Middle Triassic (e.g., Ferrante et al., 2022). The degree of speciation, triggered by relatively confined environments, was thus relatively high during the Early and Middle Triassic, a time interval which corresponds to a time of recovery of life still occurring 10 million years after the Permian–Triassic Mass Extinction (Romano et al., 2016).

## Conclusion

Based on a revision of the coelacanth material from the Besano Formation from the collection of the Paläontologisches Institut und Museum der Universität Zürich (Switzerland), the coelacanth *Ticinepomis peyeri*, previously known only by the holotype (PIMUZ T 3925), is now represented by four specimens assigned to this species. A re-examination of the holotype and the study of the new specimens make it possible to clarify several morphological, little or not understood traits on the holotype. A new species of *Ticinepomis* is recognised from material previously referred to *Ticinepomis* cf. *T. peyeri*, and named *Ticinepomis ducanensis* sp. nov. This new species is distinguished from the type species *T. peyeri* by its larger size and some morphological features of its lower jaw, especially its dentary developing as an elongated and narrow splint-like bar. This last feature is also unique among the Latimerioidei, since all other members of this clade possess a hook-shaped dentary. *T. ducanensis* sp. nov. is present both in the middle Prosanto Formation (Anisian–Ladinian boundary) at the Ducanfurgga area and in the isochronous and spatially close upper Besano Formation at Monte San Giorgio. Compared to more derived Latimeriidae, such as *Latimeria*, *Ticinepomis* presents some distinguishing characters, such as two pairs of parietals of the same length, supraorbitals as wide as the parietals and one or two first rays of the anterior dorsal fin smaller than the posterior ray. Although having a ‘conservative’ Bauplan, *Ticinepomis* presents some highly derived anatomical characters, as a lachrymojugal with a posterior triangular shape and a short body including less than 50 neural arches, features that are shared with the unusual *Foreyia* from the Middle Triassic (early Ladinian) of the Prosanto Formation (Canton Graubünden, Switzerland) and with *Rieppelia* (Ferrante & Cavin, 2023) from the Middle Triassic of Monte San Giorgio (Canton Ticino). The recognition of a new coelacanth species indicates that the specific and morphological diversity of this otherwise slow-evolving lineage of sarcopterygian fishes was particularly high in this part of the Western Tethys during the Middle Triassic, especially between 242 and 240 million years ago.

## Data Availability

The holotype of *Ticinepomis peyeri* (PIMUZ T 3925) and *Ticinepomis ducanensis* sp. nov. (PIMUZ A/I 2985) are kept together with the referred material in the collection of the Paläontologisches Institut und Museum der Universität Zürich (Canton Zürich, Switzerland).

## Abbreviations

ana.f: Anal fin
a.w.Par: Ascending wing of parasphenoid
Acl: Anocleithrum
Ang: Angular
ant.pr: Antotic process
Aup: Autopalatine
Boc: Basioccipital
Bsph: Basisphenoid
BSP: Bayerische Staatssammlung für Paläontologie und Geologie, Munich, Germany
cau.f: Caudal fin (upper/lower lobe)
Cb: Ceratobranchial
Ch: Ceratohyal
Cl: Cleithrum
Cla: Clavicle
Cor: Coronoid
De: Dentary
Dpl: Dermopalatine
D2.b: Basal plate of the posterior dorsal fin
d1.f: Anterior dorsal fin
d2.f: Posterior dorsal fin
Ecpt: Ectopterygoid
Ext: Lateral extrascapular
Gu: Gular plate
gu.p.l: Gular pit line
h.a: Haemal arches
L.j: Lachrymojugal
L.r: Lateral rostral
Mpt: Metapterygoid
m.s.c: Mandibular sensory canal
n.a: Neural arches
Op: Opercle
ot.sh: Otic shelf
p.Cor: Principal coronoid
p.m.s.c: Pore for the mandibular sensory canal
p.w.Pro: Posterior wing of the prootic
Pa: Parietal
Par: Parasphenoid
Part: Prearticular
Pmx: Premaxilla
Po: Postorbital
Pop: Preopercle
Pp: Postparietal
pr.con: Processus connectens
Preo: Preorbital
Pro: Prootic
Pt: Pterygoid
pect.f: Pectoral fin
pelv.f: Pelvic fin
PIMUZ: Paläontologisches Institut und Museum der Universität Zürich, Switzerland
Q: Quadrate
Rart: Retroarticular
Scc: Scapulocoracoid
sn.b: Snout bones
So: Supraorbital
sop.br: Subopercular branch of the preopercular canal
Spl: Splenial
Sq: Squamosal
Stt: Supratemporal
t.p: Tooth plate
t.p.Bb: Basibranchial tooth plate
UNESCO: United Nations Educational, Scientific and Cultural Organization
v.pr.Pa: Ventral descending process of the parietal
v.pr.Pp: Ventral descending process of the postparietal
v.pr.Stt: Ventral descending process of supratemporal

## References

[CR1] Alessandri G (1910). Studi sui pesci triasici della Lombardia. Memorie Societa Italiana Scienze Naturale.

[CR2] Andersson E (1916). Über einige Trias-Fische aus der Cava Trefontane. Tessin. Bull. Geol. Inst. Uppsala.

[CR3] Bassani F (1896). La ittiofauna della Dolomia Principale di Giffoni. Palaeontographia Ital..

[CR4] Bellotti C, Stoppani A (1857). Descrizione di alcune nuove specie di pesci fossili di Perledo e di altre località Lombarde. Studii Geologici e Paleontologici sulla Lombardia.

[CR5] Beltan L (1972). La faune ichthyologique du Muschelkalk de la Catalogne. Memorias De Ia Real Academia De Ciencias y Artes De Barcelona.

[CR6] Beltan L (1984). Quelques poissons du Muschelkalk superieur d'Espagne. Acta Geologica Hispanica.

[CR7] Bernasconi SM (1994). Geochemical and microbial controls on dolomite formation in anoxic environments: A case study from the Middle Triassic (Ticino, Switzerland). Contributions to Sedimentary Geology.

[CR8] Bertotti G (1991). Early Mesozoic extension and Alpine tectonics in the western Southern Alps. The geology of the area between Lugano and Menaggio (Lombardy, Northern Italy). Mem. Sci. Geol. (padova).

[CR9] Brack P, Rieber H, Nicora A, Mundil R (2005). The global boundary stratotype section and point (GSSP) of the Ladinian Stage (Middle Triassic) at Bagolino (Southern Alps, Northern Italy) and its implications for the Triassic time scale. Episodes Journal of International Geoscience.

[CR10] Bürgin T, Eichenberger U, Furrer H, Tschanz K (1991). Die Prosanto-Formation—eine fischreiche Fossil-Lagerstätte in der Mitteltrias der Silvretta-Decke (Kanton Graubünden, Schweiz). Eclogae Geologicae Helvetiae.

[CR11] Cavin L, Furrer H, Obrist C (2013). New coelacanth material from the Middle Triassic of eastern Switzerland, and comments on the taxic diversity of actinistans. Swiss Journal of Geosciences.

[CR12] Cavin L, Grădinaru E (2014). *Dobrogeria aegyssensis*, a new early Spathian (Early Triassic) coelacanth from north Dobrogea (Romania). Acta Geologica Polonica.

[CR13] Cavin L, Mennecart B, Obrist C, Costeur L, Furrer H (2017). Heterochronic evolution explains novel body shape in a Triassic coelacanth from Switzerland. Scientific Reports.

[CR14] Clément G (1999). The actinistian (Sarcopterygii) *Piveteauia madagascariensis* Lehman from the lower Triassic of northwestern Madagascar: A redescription on the basis of new material. Journal of Vertebrate Paleontology.

[CR15] Clément G (2005). A new coelacanth (Actinistia, Sarcopterygii) from the Jurassic of France, and the question of the closest relative fossil to Latimeria. Journal of Vertebrate Paleontology.

[CR16] Costa OG (1862). Studii sopra i terreni ad ittiolitti del Regno di Napoli diretti a stabilire l'eta geologica dei medesirni. Appendice Agli Atti Real Accadeimia Delle Scienze, Napoli.

[CR17] Deesri U, Cavin L, Amiot R, Bardet N, Buffetaut E, Cuny G, Giner S, Martin JE, Suan G (2018). A mawsoniid coelacanth (Sarcopterygii: Actinistia) from the Rhaetian (Upper Triassic) of the Peygros quarry, Le Thoronet (Var, southeastern France). Geological Magazine.

[CR18] Dutel H, Maisey JG, Schwimmer DR, Janvier P, Herbin M, Clément G (2012). The giant Cretaceous Coelacanth (Actinistia, Sarcopterygii) *Megalocoelacanthus dobiei* Schwimmer, Stewart and Williams, 1994, and its bearing on Latimerioidei interrelationships. PLoS ONE.

[CR19] Elliott DK (1987). A new specimen of *Chinlea sorenseni* from the Chinle Formation, Dolores River, Colorado. Journal of the Arizona-Nevada Academy of Science.

[CR20] Ferrante C, Martini R, Furrer H, Cavin L (2017). Coelacanths from the Middle Triassic of Switzerland and the pace of actinistian evolution. Research & Knowledge.

[CR21] Ferrante C, Menkveld-Gfeller U, Cavin L (2022). The first Jurassic coelacanth from Switzerland. Swiss Journal of Paleontology.

[CR30] Ferrante, C., & Cavin, L. (2023). Early Mesozoic burst of morphological disparity in the slow-evolving coelacanth fish lineage. *Scientific Reports*, 13(1), 11356. 10.1038/s41598-023-37849-9 10.1038/s41598-023-37849-9 PMC10345187 37443368

[CR22] Forey PL (1998). History of the Coelacanth Fishes.

[CR23] Frauenfelder A (1916). Beiträge zur Geologie der Tessiner Kalkalpen. Eclogae Geologicae Helvetiae.

[CR24] Furrer H (1995). The Kalkschieferzone (Upper Meride Limestone; Ladinian) near Meride (Canton Ticino, Southern Switzerland) and the evolution of a Middle Triassic intraplatform basin. Eclogae Geologicae Helvetiae.

[CR25] Furrer, H. (2003). Der Monte San Giorgio im Südtessin—vom Berg der Saurier zur Fossil-Lagerstätte internationaler Bedeutung. *Neujahrsblatt der Naturforschenden Gesellschaft in Zürich, 206,* 64 pp.

[CR26] Furrer, H. (2019). Fische und Saurier aus dem Hochgebirge. Fossilien aus der mittleren Trias bei Davos. *Neujahrsblatt der Naturforschenden Gesellschaft in Zürich, 221*, 112 pp.

[CR27] Furrer H, Froitzheim U, Wurster D (1992). Geologie, Stratigraphie und Fossilien der Ducankette und des Landwassergebiets (Silvretta-Decke, Ostalpin). Eclogae Geologicae Helvetiae.

[CR28] Furrer H, Schaltegger U, Ovtcharova M, Meister P (2008). U-Pb zircon age of volcaniclastic layers in Middle Triassic platform carbonates of the Austroalpine Silvretta nappe (Switzerland). Swiss Journal of Geosciences.

[CR29] Furrer, H., & Vandelli, A. (2014). *Guide to the Museum of fossils from Monte San Giorgio Meride. Fondazione del Monte San Giorgio*. Fondazione del Monte San Giorgio, Mendrisio, 128 pp.

[CR31] Gall JC, Grauvogel L, Lehman JP (1974). Faune du Buntsandstein. V. Les Poissons Fossiles de la collection Grauvogel-Gall. *Annales de Paléontologie (Vertébrés)*. Paris.

[CR32] Geng BH, Zhu M, Jin F (2009). A revision and phylogenetic analysis of *Guizhoucoelacanthus* (Sarcopterygii, Actinistia) from the Triassic of China. Vertebrata PalAsiatica.

[CR33] Gess RW, Coates MI (2015). Fossil juvenile coelacanths from the Devonian of South Africa shed light on the order of character acquisition in actinistians. Zoological Journal of the Linnean Society.

[CR34] Hagdorn H, Mutter RJ (2011). The vertebrate fauna of the Lower Keuper Albertibank (Erfurt Formation, Middle Triassic) in the vicinity of Schwäbisch Hall (Baden-Württemberg, Germany). Palaeodiversity.

[CR35] Hartung J, Sander PM, Friedman M, Wintrich T (2021). First record of mawsoniid coelacanths (Actinistia, Sarcopterygii) from the marine Rhaetian (Upper Triassic) of Bonenburg. Germany. Journal of Vertebrate Paleontology.

[CR36] Hensel K (1986). Morphologie et interprétation des canaux et canalicules sensoriels céphaliques de *Latimeria chalumnae* Smith, 1939 (Osteichthyes, Crossopterygii, Coelacanthiformes). Bulletin Du Muséum National D'histoire Naturelle. Section a, Zoologie, Biologie Et Écologie Animales.

[CR37] Hunt AP (1997). A new coelacanth (Osteichthyes: Actinistia) from the continental Upper Triassic of New Mexico. New Mexico Museum of Natural History and Science Bulletin.

[CR38] Johnson SC, Lucas SG, Hunt AP, Heckert AB (2002). Macro-fish fauna of the Upper Triassic (Apachean) Redonda Formation, eastern New Mexico. Upper Triassic Stratigraphy and Paleontology, New Mexico Museum of Natural History Bulletin.

[CR39] Kner R (1866). Die Fische der bituminösen Schiefer von Raibl in Kärnthen. *Sitzungsberichte der Akademie der Wissenschaften*. Mathematisch Naturwissenschaftliche Classe. Wien.

[CR40] Kuhn-Schnyder E (1974). Die Triasfauna der Tessiner Kalkalpen. Neujahrsblatt Der Naturforschenden Gesellschaft Zürich.

[CR41] Lambers, P. (1992).* On the Ichthyofauna of the Solnhofen Lithographic Limestone (Upper Jurassic, Germany)*. PhD Thesis. pp. 336.

[CR42] López-Arbarello A, Stockar R, Bürgin T (2014). Phylogenetic relationships of the Triassic Archaeosemionotus Deecke (Halecomorphi, Ionoscopiformes) from the ‘Perledo Fauna’. PLoS ONE.

[CR43] Lund R, Lund WL (1985). Coelacanths from the Bear Gulch Limestone (Namurian) of Montana and the evolution of the Coelacanthiformes. Bulletin of Carnegie Museum of Natural History.

[CR44] Martin M, Wenz S (1984). Découverte d'un nouveau Coelacanthide, Garnbergia ommata ng, n. sp., dans le Muschelkalk supérieur du Baden-Württemberg. *Stuttgarter Beiträge zur Naturkunde*. Serie B (geologie Und Paläontologie).

[CR45] Meunier FJ, Cupello C, Yabumoto Y, Brito PM (2018). The diet of the Early Cretaceous coelacanth† *Axelrodichthys araripensis* Maisey, 1986 (Actinistia: Mawsoniidae). Cybium.

[CR46] Meunier FJ, Mondéjar-Fernández J, Goussard F, Clément G, Herbin M (2015). Presence of plicidentine in the oral teeth of the coelacanth *Latimeria chalumnae* Smith 1939 (Sarcopterygii; Actinistia). Journal of Structural Biology.

[CR47] Millot J, Anthony J (1958). Anatomie de Latimeria chalumnae—Tome I: squelette, muscles et formations de soutien.

[CR48] Mundil R, Pálfy J, Renne PR, Brack P (2010). The Triassic timescale: New constraints and a review of geochronological data. Geological Society, London, Special Publications.

[CR49] Muttoni G, Nicora A, Brack P, Kent DV (2004). Integrated Anisian-Ladinian boundary chronology. Palaeogeography, Palaeoclimatology, Palaeoecology.

[CR50] Peyer B (1944). Die Reptilien vom Monte San Giorgio. Neujahrsblatt Der Naturforschenden Gesellschaft Zürich.

[CR51] Pfiffner A (2014). Geology of the Alps.

[CR52] Preto N, Kustatscher E, Wignall PB (2010). Triassic climates—state of the art and perspectives. Palaeogeography, Palaeoclimatology, Palaeoecology.

[CR53] Reis OM (1900). *Coelacanthus lunzensis* Teller. Jahrbuch Der Kaiserlich-Königlichen Geologischen Reichsanstalt.

[CR54] Renesto S, Kustatscher E (2019). A coelacanth fish from the Anisian (Middle Triassic) of the Dolomites. Rivista Italiana Di Paleontologia e Stratigrafia.

[CR55] Renesto S, Magnani F, Stockar R (2021). A new coelacanth specimen with elongate ribs from the Middle Triassic (Ladinian) Kalkschieferzone of Monte San Giorgio (Canton Ticino, Switzerland). Rivista Italiana Di Paleontologia e Stratigrafia.

[CR56] Renesto S, Stockar R (2018). First record of a coelacanth fish from the Middle Triassic Meride Limestone of Monte San Giorgio (Canton Ticino, Switzerland). Rivista Italiana Di Paleontologia e Stratigrafia.

[CR57] Rieber H (1973). Cephalopoden aus der Grenzbitumenzone (Mittlere Trias) des Monte San Giorgio (Kanton Tessin, Schweiz). Mémoires Suisses De Paléontologie.

[CR58] Rieppel O (1980). A new coelacanth from the Middle Triassic of Monte San Giorgio, Switzerland. Eclogae Geologicae Helvetiae.

[CR59] Rieppel O (1985). A second actinistian from the Middle Triassic of Monte San Giorgio, Kanton Tessin. Switzerland. Eclogae Geologicae Helvetiae.

[CR60] Röhl HJ, Schmid-Röhl A, Furrer H, Frimmel A, Oschmann W, Schwark L (2001). Microfacies, geochemistry and palaeoecology of the Middle Triassic Grenzbitumenzone from Monte San Giorgio (Canton Ticino, Switzerland). Geologia Insubrica.

[CR61] Romano C, Koot MB, Kogan I, Brayard A, Minikh AV, Brinkmann W, Bucher H, Kriwet J (2016). Permian-Triassic Osteichthyes (bony fishes): Diversity dynamics and body size evolution. Biological Reviews.

[CR62] Schaeffer B (1952). The Triassic coelacanth fish *Diplurus*, with observations on the evolution of the Coelacanthini. Bulletin of the AMNH.

[CR63] Schaeffer B (1967). Late Triassic fishes from the western United States. Bulletin of the AMNH.

[CR64] Schaeffer B, Gregory JT (1961). Coelacanth fishes from the continental Triassic of the western United States. American Museum Novitates.

[CR65] Schaumberg G (1978). Neubeschreibung von Coelacanthus granulatus Agassiz (Actinistia, Pisces) aus dem Kupferschiefer von Richelsdorf (Perm, W.-Deutschland). Paläontologische Zeitschrift.

[CR66] Schultze H-P (1980). Eier legende und lebendgebärende Quastenflosser. Natur Und Museum.

[CR67] Schweizer R (1966). Ein Coelacanthide aus dem Oberen Muschelkalk Göttingens. Neues Jahrbuch Für Geologie Und Paläontologie, Abhandlungen.

[CR68] Stensiö EA (1921). Triassic fishes from Spitzbergen. Part 1.

[CR69] Stensiö EA (1932). Triassic fishes from East Greenland: Collected by the Danish expeditions in 1929–1931. Meddelelser Om Grønland, Copenhagen.

[CR70] Stockar R, Adatte T, Baumgartner PO, Föllmi KB (2013). Palaeoenvironmental significance of organic facies and stable isotope signatures: The Ladinian San Giorgio Dolomite and Meride Limestone of Monte San Giorgio (Switzerland, WHL UNESCO). Sedimentology.

[CR71] Stockar R, Baumgartner PO, Condon D (2012). Integrated Ladinian bio-chronostratigraphy and geochrononology of Monte San Giorgio (Southern Alps, Switzerland). Swiss Journal of Geosciences.

[CR72] Teller, F. (1891) Über den Schädel eines fossilen Dipnoërs *Ceratodus sturii* nov. spec. aus den Schichten der oberen Trias der Nordalpen. *Abhandlungen der Kaiserlich-Königlichen Geologischen Reichsanstalt. Vienna, 15*(3), 1–39.

[CR73] Toriño P, Soto M, Perea D (2021). A comprehensive phylogenetic analysis of coelacanth shes (Sarcopterygii, Actinistia) with comments on the composition of the Mawsoniidae and Latimeriidae: evaluating old and new methodological challenges and constraints. Historical Biology.

[CR74] Uyeno T, Tsutsumi T, Musick JA, Bruton MN, Balon EK (1991). Stomach contents of Latimeria chalumnae and further notes on its feeding habits. The biology of Latimeria chalumnae and evolution of coelacanths.

[CR75] Vérard C (2019). Panalesis: Towards global synthetic palaeogeographies using integration and coupling of manifold models. Geological Magazine.

[CR76] Wen W, Zhang QY, Hu SX, Benton MJ, Zhou CY, Tao, Huang JY, Chen ZQ (2013). Coelacanths from the Middle Triassic Luoping Biota, Yunnan, South China, with the earliest evidence of ovoviviparity. Acta Palaeontologica Polonica.

[CR77] Witzmann F, Dorka M, Korn D (2010). A juvenile early Carboniferous (Viséan) coelacanth from Rösenbeck (Rhenish Mountains, Germany) with derived postcranial characters. Fossil Record.

[CR78] Zatoń M, Broda K, Qvarnström M, Niedźwiedzki G, Ahlberg PE (2017). The first direct evidence of a Late Devonian coelacanth fish feeding on conodont animals. The Science of Nature.

